# Integrating cell interaction with transcription factors to obtain a robust gene panel for prognostic prediction and therapies in cholangiocarcinoma

**DOI:** 10.3389/fgene.2022.981145

**Published:** 2022-11-30

**Authors:** Tingjie Wang, Chuanrui Xu, Dan Xu, Xiaofei Yang, Yaxin Liu, Xiujuan Li, Zihang Li, Ningxin Dang, Yi Lv, Zhijing Zhang, Lei Li, Kai Ye

**Affiliations:** ^1^ National Local Joint Engineering Research Center for Precision Surgery and Regenerative Medicine, Shaanxi Provincial Center for Regenerative Medicine and Surgical Engineering, Center for Mathematical Medical, The First Affiliated Hospital of Xi’an Jiaotong University, Xi’an, China; ^2^ School of Pharmacy, Tongji Medical College, Huazhong University of Science and Technology, Wuhan, China; ^3^ School of Life Science and Technology, Xi’an Jiaotong University, Xi’an, Shaanxi, China; ^4^ School of Computer Science and Technology, Faculty of Electronic and Information Engineering, Xi’an Jiaotong University, Xi’an, Shaanxi, China; ^5^ Genome Institute, The First Affiliated Hospital of Xi’an Jiaotong University, Xi’an, Shaanxi, China; ^6^ MOE Key Lab for Intelligent Networks and Networks Security, Faculty of Electronic and Information Engineering, Xi’an Jiaotong University, Xi’an, Shaanxi, China; ^7^ School of Automation Science and Engineering, Faculty of Electronic and Information Engineering, Xi’an Jiaotong University, Xi’an, Shaanxi, China; ^8^ Faculty of Science, Leiden University, Leiden, Netherlands

**Keywords:** cell communication, single cell RNAseq, transcript factor, cholangiocarcinoma, immunotherapy

## Abstract

**Objective:** The efficacy of immunotherapy for cholangiocarcinoma (CCA) is blocked by a high degree of tumor heterogeneity. Cell communication contributes to heterogeneity in the tumor microenvironment. This study aimed to explore critical cell signaling and biomarkers induced *via* cell communication during immune exhaustion in CCA.

**Methods:** We constructed empirical Bayes and Markov random field models eLBP to determine transcription factors, interacting genes, and associated signaling pathways involved in cell-cell communication using single-cell RNAseq data. We then analyzed the mechanism of immune exhaustion during CCA progression.

**Results:** We found that *VEGFA*-positive macrophages with high levels of *LGALS9* could interact with *HAVCR2* to promote the exhaustion of CD8^+^ T cells in CCA. Transcription factors *SPI1* and *IRF1* can upregulate the expression of *LGALS9* in *VEGFA*-positive macrophages. Subsequently, we obtained a panel containing 54 genes through the model, which identified subtype S2 with high expression of immune checkpoint genes that are suitable for immunotherapy. Moreover, we found that patients with subtype S2 with a higher mutation ratio of *MUC16* had immune-exhausted genes, such as *HAVCR2* and *TIGIT.* Finally, we constructed a nine-gene eLBP-LASSO-COX risk model, which was designated the tumor microenvironment risk score (TMRS).

**Conclusion:** Cell communication-related genes can be used as important markers for predicting patient prognosis and immunotherapy responses. The TMRS panel is a reliable tool for prognostic prediction and chemotherapeutic decision-making in CCA.

## 1 Introduction

Cholangiocarcinoma (CCA) is the second most common form of liver malignancy with increasing diagnostic incidence and mortality rates (Banales et al., 2020). Patients with CCA are divided into two subtypes depending on their anatomical site of origin: intrahepatic (iCCA or ICC), perihilar (pCCA), and distal (dCCA) CCA (Bridgewater et al., 2014). Most patients with CCA have lost the opportunity to undergo surgical resection upon diagnosis (Brandi et al., 2020). Recent studies have revealed that immunotherapies can protect against tumor development and metastasis. Immune checkpoint inhibitors (ICIs), chimeric antigen receptor T-cells, and tumor vaccines are the main immunotherapies used in clinical practice.

Tumor heterogeneity and stromal/immune cells in the tumor immune microenvironment (TME) significantly influence the effect of the immunotherapy (Tang et al., 2021). Tumors can be categorized into inflamed, immune desert, or immune-excluded phenotypes based on the spatial localization of immune cells in the tumor and stromal compartments (Hegde and Chen, 2020). Inflamed tumors are typically associated with a response to ICIs, particularly PD-L1- and PD-1-directed antibodies (Jiang et al., 2020). The clinical efficacy of PD-1/PD-L1 ICIs enhance the cytotoxicity of CD8^+^ T cells. However, clinical experience has shown that a large percentage of CCAs (>50%) do not respond to ICIs (e.g., high tumor PD-L1 expression) (Hegde and Chen, 2020). Therefore, it is necessary to investigate the components of TME to identify other immune checkpoints during CCA immunotherapy.

Interactions between ligands and receptors in the TME and tumor cells play a crucial role in tumor progression (Zhang et al., 2020). It has been revealed that cancer-associated fibroblasts (CAF) in ICC induced significant epigenetic alterations in ICC cells *via* IL-6 secretion and subsequent upregulation of *EZH2* through *STAT3* activation (Zhang et al., 2020). In addition, PD-L1+ TAMs in the TME facilitate CCA progression (Rizzo et al., 2021a). However, the mechanism of cell interaction and upstream and downstream signal transduction elements influencing the CCA immunotherapy of remains unclear.

In the present study, we developed an eLBP algorithm by integrating empirical Bayes with Markov random fields to infer the intact intercellular communication pattern, including interacting cell types and critical TFs that could promote cell interactions simultaneously. We revealed a unique signaling transduction model between tumor-associated macrophages and exhausted CD8^+^ T cells that could influence immunotherapy and novel immune checkpoint genes in CCA patients. We also constructed a gene classifier to distinguish inflamed tissue with high expression of immune checkpoint genes (ICGs) from the immune desert subtype and predict the immunotherapeutic effect in the two CCA subtypes.

## 2 Materials and methods

### 2.1 Bulk RNA-seq and single-cell datasets

CCA sequencing data were retrieved from the Gene Expression Omnibus (GEO) database (https://www.ncbi.nlm.nih.gov/). All the datasets used in this study are listed in Supplementary Table S1.

### 2.2 Single-cell RNA-seq data processing

The single-cell datasets were downloaded from the GEO database (Supplementary Table S1). The raw gene expression matrix was imported and processed using the Seurat R package (version 3.1.5) (Butler et al., 2018). Cells with unique molecular identifier counts below 200 or mitochondrial content above 35% were removed. Normalization and dimension-reduction processes were performed using Seurat R package (3.1.5). Clusters were computed using the FindClusters function (resolution = 0.8) and visualized using uniform manifold approximation and projection (UMAP), as implemented in Seurat. Differential expression between clusters was calculated using a likelihood-ratio test for single-cell gene expression implemented in Seurat, with a family-wise error rate of 5%. Cell types were defined according to lineage-specific marker genes.

### 2.3 Empirical bayes loopy belief propagation algorithm

Interactions are frequent among TME cells depending on the ligand and receptor pairs. We evaluated the contribution of cell-interaction genes to cell communication by measuring the importance of their participant-enriched pathways in each cell type. Meanwhile, transcription factors can enhance cell signaling by promoting the expression of interacting genes. The importance and critical TF regulons were calculated using empirical Bayes loopy belief propagation (eLBP). The cell clustering results and differential genes in each cell type were used as the input data for eLBP. The algorithm consists of the following five steps based on cell interaction genes (CIGs).

#### 2.3.1 Construction of interaction network of CIGs

We first identified the differentially expressed genes (DEGs) for each cell type. The pathways that were significantly enriched (*P*.adj <0.05) in the KEGG, GO, and REACTOME datasets for each cell type were selected. We obtained all potential interaction gene pairs during cell communication from the CellChat database (Jin et al., 2021). Next, we extracted the pathways containing cell-communication genes in each cell type. Subsequently, we conducted the protein interaction (Tohonen et al., 2015) analysis of these pathway genes to produce a potential gene interaction undirected graph for each cell type.

#### 2.3.2 Construction of a Markov random field for CIGs

After constructing the gene interaction undirected graph for each cell type, we adopted an empirical Bayes Markov random field (MRF) network to calculate the importance of communication-related genes in each cell type. The network was represented by $G_{k}=\left\{V^{k},\;E^{k}\right\}$ in where k∈{1,2,…,N} denotes the cell types obtained from a single-cell dataset, $V_{i}^{k}=\in \left\{V_{1}^{k}\;,V_{2}^{k},\ldots ,V_{M}^{k}\right\}$ denotes the gene nodes in the undirected graph in the cell type *k* containing total *M* genes, and $E=\left\{\left(V_{i}^{k},\;V_{j}^{k}\right),\;V_{i}^{k},\;V_{j}^{k}\in V\right\}$ represents the interaction intensity between two genes *V*_*i*_ and *V*_*j*_ in the interaction network of this cell type *k*. In addition, we set the dummy variable L={+1,−1} to denote a set of labels, in which +1 denotes the genes that were present in the cell type *k*, while −1 indicates the genes absent in the cell type *k*.

To construct a complete MRF model, we specified the node potentials of the class labels of node $V_{i}^{k}$ , denoted as $\psi \left(V_{i}^{k}\right)$ , which are denoted as *V*_*i*_, and *V*_*j*_ are denoted as $\psi \left(V_{i}^{k},\;V_{j}^{k}\right)$ . In this formulation, we defined node potentials as the probabilities that genes could occur in the cell type *k*, $\psi \left(V_{i}^{k}\right)=P\left(V_{i}^{k}\right)$ . Moreover, we defined the cosine similarity, $Co\;\sin\left(V_{i}^{k},\;V_{j}^{k}\right)$ , between two adjacent nodes with labels $V_{i}^{k}$ and $V_{j}^{k}$ as the edge potential. We define potential functions and calculated the joint distribution of all indicator variables *V* can be denoted as Eq. 1 (Song You et al., 2018).

$$\begin{array}{l} p\left(V\right)=\frac{1}{Z}\prod_{V_{i}^{k}\in V}\psi \left(V_{i}^{k}\right)\prod_{\left(V_{i}^{k},\;V_{j}^{k}\right)\in E}\psi \left(V_{i}^{k},\;V_{j}^{k}\right)\\ \left(i,\;j\in \left\{1,\;2,\ldots ,M\right\};\;k\in \left\{1,\;2,\ldots ,N\right\}\right) \end{array}$$ (1)

where, *Z* is the normalization constant. Next, we obtained the eLBP, a widely adopted approximate inference algorithm for the MRF. When the eLBP converges, the final belief node $V_{i}^{k}$ can be calculated using Eq. 2 (Tirosh et al., 2016).

$$\begin{array}{l} S\left(V_{i}^{k}\right)=\frac{1}{Z_{2}}\psi \left(V_{i}^{k}\right)\prod_{V_{j}^{k}\in N_{i}}m_{j\to i}\left(V_{i}^{k}\right)\\ \left(i,j\in \left\{1,\;2,\ldots ,M\right\};\;k\in \left\{1,\;2,\ldots ,N\right\}\right) \end{array}$$ (2)

where *Z*_2_ is the normalization constant, and *N*_*i*_ denotes the neighbor nodes connected with node *V*_*i*_ in cell type *k*.

#### 2.3.3 Construction of positive and negative reference cells and calculation of the prior probability of each gene node in eLBP

The prior probabilities in Eqs 1, 2 are calculated using the empirical Bayes method. The prior probability of node $V_{i}^{k}$ in the network indicates the probability of the gene *i* being presented in a cell type *k*. To calculate the prior probabilities of genes in cell-type *k*, we first constructed a cell-type *k* positive and negative dataset. Here, we first removed the genes whose expression levels were equal to zero in more than 50% of the cells in each type. Then, we randomly selected 200 or all the cells if the cell number was <200 to obtain a positive dataset of cell type *k*, we selected 200 cells that were not in cell type *k* to obtain the corresponding negative dataset.

We then assumed that genes in the cell type *k* followed a Poisson distribution with the parameter *θ* (Eq. 3).

$$P\left(V|\theta \right)=e^{-\theta }\theta^{v}/v!$$ (3)

where *θ* has a Gamma prior with unknown parameters *α* and *β* (Eq. 4).

$$\pi \left(\theta \right)=\frac{\beta^{\alpha }}{\Gamma \left(\alpha \right)}e^{-\beta \theta }\theta^{\alpha -1}$$ (4)

Based on this assumption, under the observation vector *X*_*CN*_, we calculated the posterior values of *α* and *β via* Eq. 5 by randomly sampling 50% of the cells in *X*_*CN*_.

$$E\left(x\right)=\frac{\alpha }{\beta };\;Var\left(x\right)=\frac{\alpha \left(1+\beta \right)}{\beta^{2}}$$ (5)

Subsequently, we calculated the posterior mean of $\theta_{iL}^{k}$ using Eq. 6, using the other data in *X*_*CN*_.

$$\begin{array}{l} \theta_{iL}^{k}=E\left(\frac{x_{i}+\alpha }{1+\beta }\right)\\ \left(L\in \left\{+1,-1\right\};\;k\in \left\{1,\;2,\ldots ,N\right\};\;i\in \left\{1,\;2,\ldots ,M\right\}\right) \end{array}$$ (6)

where *L* denotes the cells from the positive (+1) or negative datasets (−1). Next, we calculated the prior probability of node $V_{i}^{k}(\psi \left(V_{i}^{k}\right)$ using Eq. 7.

$$\begin{array}{l} \psi \left(V_{i}^{k}\right)=\left\{ \begin{array}{l} \frac{\theta_{iL=+1}^{k}}{\theta_{iL=+1}^{k}+\theta_{iL=-1}^{k}},\;if\;L_{i}=+1\\ \frac{\theta_{iL=-1}^{k}}{\theta_{iL=-1}^{k}+\theta_{iL=+1}^{k}},\;if\;L_{i}=-1 \end{array}\right.\\ \left(k\in \left\{1,\;2,\ldots N\right\};\;i\in \left\{1,\;2,\ldots ,M\right\}\right) \end{array}$$ (7)

Finally, we calculated the probability of occurrence of gene nodes in the eLBP network using Eq. 2.

#### 2.3.4 Calculation of cell interaction score

After obtaining the posterior probability of each gene node, we obtained a simplified interaction network by trimming the gene nodes whose posterior probability obtained from Eq. 2 was <0.7. We then calculated the connection strength among the CIG pairs between cell types *K*_*i*_ and *K*_*j*_ (CGscore) using Eq. 8.

$$CG_{score_{K_{i}K_{j}}}=S\left(V_{i}^{k}\right)*Co\sin\left(V_{i}^{k}*V_{j}^{k}\right)*S\left(V_{j}^{k}\right);\;k\in \left(1,\;N\right);\;i,j\in \left(1,\;M\right)$$ (8)

And the cell type interaction strength CT score was calculated using Eq. 9.

$$CT_{score_{ij}}=mean\left(CG_{score_{K_{i}K_{j}}}\right);\;k\in \left(1,\;N\right);\;i,j\in \left(1,\;M\right)$$ (9)

where *K*_*i*_ and *K*_*j*_ indicate different cell types. The highest CT score indicates potentially interacting cell types.

#### 2.3.5 Construction of transcription factor -cell interaction signal transduction pathway

In addition to the cell interaction community, we calculated the transcription factor (TF) regulons that could enhance the critical cell-interaction genes in the eLBP model. To achieve this, we first obtained the potential regulatory networks of TFs by collating three transcription factor databases: ENCODE, ChEA3, and TRANSFAC. Next, we obtained the TFs whose target genes were significantly enriched in the trimmed eLBP network obtained from step 4 in the three TF datasets (ChEA3, ENCODE, and TRANSFAC, *p* < 0.05). Subsequently, we calculated the contribution score of each TF using Eq. 10:

$$TF_{score_{q}}=mean\left(S\left(V_{i}^{k}\right)*Co\sin\left(V_{i}^{k},\;TF_{q}\right)\right);\;k\in \left(1,\;N\right);\;i,j\in \left(1,\;M\right)$$ (10)

where $V_{i}^{k}$ indicates the gene node *i* in cell type *k* in eLBP and *TF*_*q*_, and $Co\sin\left(V_{i}^{k},\;TF_{q}\right)$ represents the cosine similarity between gene node *V*_*i*_ and *TF*_*q*_. Ultimately, we obtained the strongest interactive cell types, important interactive gene pairs, and key TF regulons that could promote CIGs.

### 2.4 THP-1 cell culture and macrophage differentiation

Human leukemia monocytic THP-1 cells were cultured in Gibco™ RPMI 1640 medium supplemented with 10% fetal bovine serum (FBS), 100 units/ml penicillin, and 100 g/ml streptomycin. THP-1 cells were seeded at a density of 6 × 10^5^ cells/well into 24-well plates and treated with 200 ng/ml phorbol12-myristate13-acetate (PMA; Sigma-Aldrich, St. Louis, MO, United States) for 48 h. The cells were cultured at 37°C in a humidified atmosphere with 5% CO_2_, where THP-1 monocytes were differentiated into macrophages.

### 2.5 Small interfering RNA transfection

Small interfering RNA (siRNAs) against *IRF1* and *SPI1* were used to validate the regulation of the expression of *LAGLS9* in THP-1 cells. *IRF1* and *SPI1* siRNAs were designed and synthesized by GenePharma (Shanghai, China). THP-1 cells were transfected with GP-transfect-Mate, according to the manufacturer’s instructions. Briefly, THP-1 cells were seeded at a density of 6 × 10^5^ cells/well in 24-well plates and treated with 200 ng/ml for 48 h. GP-transfect-Mate was diluted in Opti-MEM (50 ml; Invitrogen, Waltham, MA, United States) for 5 min before mixing with an equal volume of Opti-MEM containing siRNA (40 pmol). After 20 min of incubation, 100 μL of the resulting GP-transfect-Mate/siRNA mixture was added directly to the cells. After 24 h of incubation at 37°C in a 5% CO_2_ atmosphere, cells were harvested for real-time PCR. The sequences of the primers used were as follows: SPI1-siRNA: CAG​GCA​GCA​AGA​AGA​AGA​UTT and AUC​UUC​UUC​UUG​CUG​CCU​T. IRF1-siRNA: GGG​CUC​AUC​UGG​AUU​AAU​ATT, UAU​UAA​UCC​AGA​UGA​GCC​CTT.

### 2.6 RNA isolation and real-time PCR

Total RNA was isolated using TRIzol reagent (Sigma-Aldrich), according to the manufacturer’s instructions. cDNA was synthesized using 5× All-in-One RT-Master Mix (Abm, Canada). Real-time PCR was performed in a LightCycler 96 using 2× chemQSYBR QPCR Master Mix (Vezyme, China), according to the manufacturer’s instructions, in a total volume of 10 μL. The sequences of the primers used were SPI1-Fw and GCGTGCAAAATGGAAG GGTTT. SPI1-Rev: GGT​ATC​GAG​GAC​GTG​CAT​CT. IRF1-Fw, GCT​GGG​ACA​TCA​ACA​AGG​AT. IRF1-Rev: CCT​GCT​CTG​GTC​TTT​CAC​CT; LGALS9-Fw: AAG​GTG​ATG​GTG​AAC​GGG​AT. LGALS9-Rev: ACT​GTC​TGG​GTA​ATG​GGA​GC; CD68-Fw: TCC​AGG​GAA​GCT​GTG​AGG​GT. CD68-Rev: AGC​CGA​GAA​TGT​CCA​CTG​TGC. CD163-Fw: TTT​GTC​AAC​TTG​AGT​CCC​TTC​AC. CD163-Rev: TCC​CGC​TAC​ACT​TGT​TTT​CAC. VEGFA-Fw: TCC​TCA​CAC​CAT​TGA​AAC​CA. VEGFA-Rev: TTT​TCT​CTG​CCT​CCA​CAA​TG.

### 2.7 Identification of CCA subclasses

Genes from eLBP were selected for consistent clustering using the R software package ConsensusClusterPlus to sort the immune molecular CCA subtypes (Wilkerson & Hayes, 2010). Correlations between subtypes, clinical features, immunity, and prognosis were analyzed.

### 2.8 Multi-omics data acquisition and processing

The somatic mutation data of all patients with CCA was obtained from the International Cancer Genome Consortium (ICGC, https://dcc.icgc.org/). Mutations were analyzed and visualized using maftools (Mayakonda et al., 2018). The enrichment scores of the hallmark genes were evaluated through single-sample GSEA (ssGSEA) using the “GSVA” R package (Hänzelmann et al., 2013). Hallmark gene sets were obtained from MSigDB (https://www.gsea-msigdb.org/gsea/msigdb/).

### 2.9 Differentially expressed gene analysis

The “limma” package was used to perform differentially expressed gene (DEG) analysis. An empirical Bayesian method was applied to estimate the DEGs between two clusters, which were identified using a consistent clustering method based on moderated *t*-tests (Ritchie et al., 2015). The adjusted *p*-value for multiple testing was calculated using the Benjamini-Hochberg correction. Genes with an absolute log_2_ (fold change) greater than one and false discovery rate (FDR) < 0.05 were identified as DEGs between the two subtypes.

### 2.10 Estimation of immune infiltration and tumor purity

We downloaded the “CIBERSORT” scripts (https://cibersort.stanford.edu/) to estimate the immune composition of patients with CCA using a normalized express matrix (Chen et al., 2018). Immune, stromal, and tumor purity scores were calculated using the “estimate” R package (Yoshihara et al., 2013).

### 2.11 DNA methylation analysis

Differential DNA methylation analysis was conducted on both normal and tumor tissues, as well as on the two tumor subtypes. Data were pre-processed using the “minfi” package (Aryee et al., 2014). Hypomethylation probes with β < 0.5 hypo-methylation probes were used to conduct the analysis. We considered CpG probes to be hypo-methylated if they met the following criteria (Song You et al., 2018): β < 0.5 (Tirosh et al., 2016); *M*-value was significantly different between the two subtypes (q-value < 0.05); and (Stuart et al., 2019) mean β-value difference (Δβ) of >0.2 between the two groups. Copy number profiles were derived from the signal intensity values of methylated and unmethylated probes using the “conumee” package in R (http://bioconductor.org/packages/conumee). Copy number variations (CNVs) were derived from the log2-ratios of the tumor samples to the average value of matched normal tissues.

### 2.12 Statistical analysis

All computational and statistical analyses were performed using R software (https://www.r-project.org/). The unpaired Student’s *t*-test was used to compare two groups with normally distributed variables, while the Mann-Whitney U-test was used to compare two groups with non-normally distributed variables. Survival analysis was performed using the Kaplan–Meier “survival” R package. The log-rank test was used to determine whether the survival curves were significantly different. A *p*-value < 0.05 was considered statistically significant.

### 2.13 Cox proportional hazards regression analysis

We used the least absolute shrinkage and selection operator (LASSO) method with 10-fold cross-validation and the Cox proportional hazards model with Akaike information criterion (AIC) selection criteria to build a nine-gene prognostic risk model based on the 54 TF-CIGs using the “glmnet” R package. The tumor mutation-burden-related signature (TMBRS) was calculated using Eq. 11:

$$TMBRS=\sum \left(\beta_{i}\times EXPi\right)$$ (11)

where β_i_ can be derived from multivariate Cox analysis. The prognostic accuracy of the classifiers in the training and testing sets was evaluated using the Kaplan–Meier curve and log-rank test.

### 2.14 Immunotherapeutic analysis

We predicted the IC_50_ values for the CCAs in GSE89749 *via* OncoPredict software using the 54 genes obtained from the eLBP algorithm (Maeser et al., 2021). In this step, we adopted the gene expression matrix from Genomics of Drug Sensitivity in Cancer (https://www.cancerrxgene.org/) and acquired drug sensitivity scores for the two subtypes. Next, we conducted a differential analysis of drugs between the two subtypes using the Wilcoxon rank-sum test. Drugs with *p*-values < 0.001 and drug sensitivity scores <1 were considered potential candidates.

## 3 Results

### 3.1 Distinct stromal cell compositions in adjacent and CCA tissues

**FIGURE 1:**
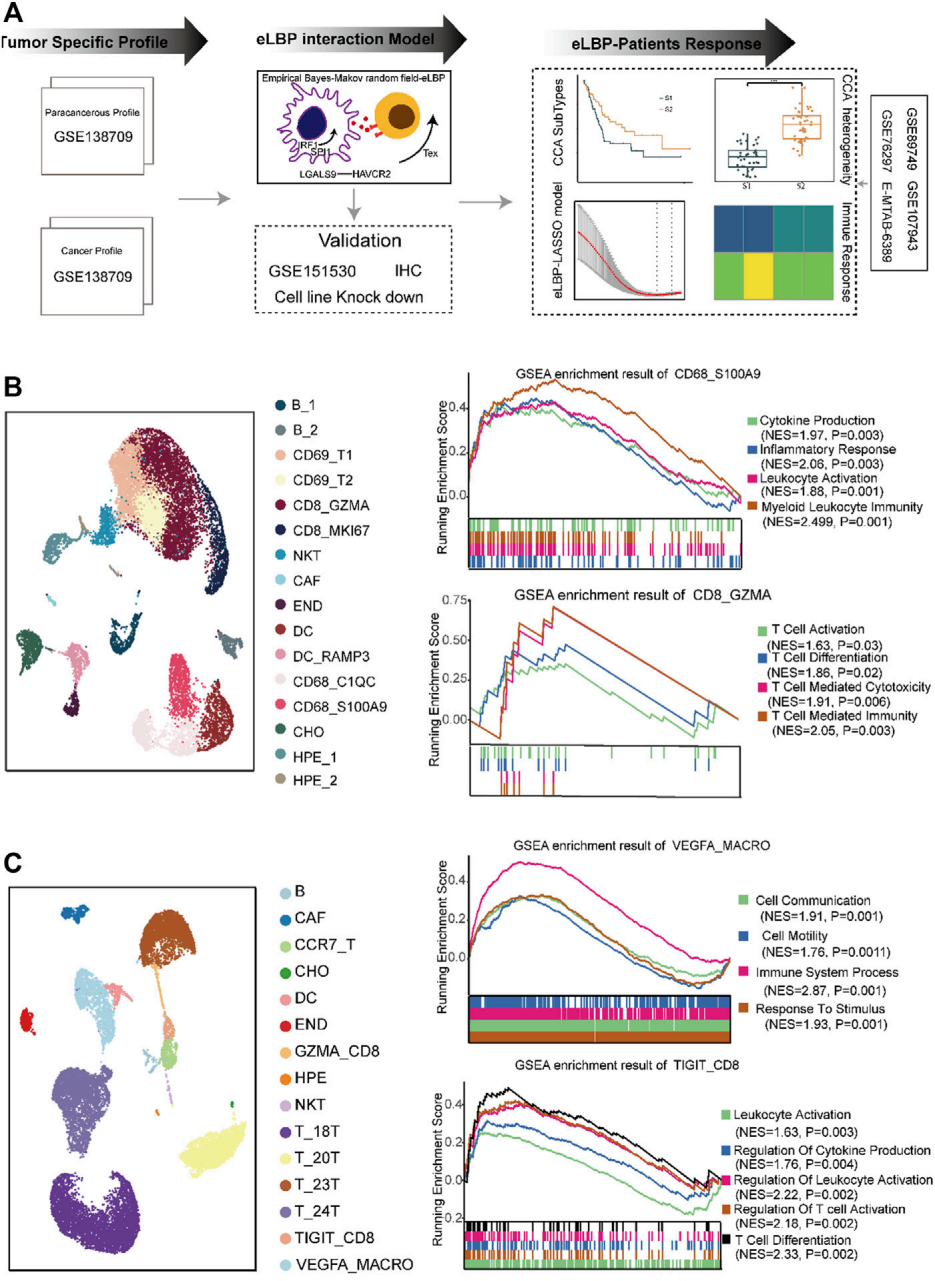
The distinct composition and function of cell types in the tumor microenvironment of cholangiocarcinoma. **(A)** Overview of the study design. We first analyzed distinct cell composition and function of the normal and cancer tissue in cholangiocarcinoma (CCA) single-cell RNAseq dataset (GSE138709). Next, we developed a method integrating empirical Bayes and Markov random field models (eLBP) which could simultaneously calculate transcription factors, interaction genes, and associated signaling pathways involved in cell-cell communication. We found that TFs *IRF1/SPI1* in *VEGFA*-positive macrophages could enhance the expression of *LGALS9* and induce the exhaustion of CD8 T cells *via LGALS9/HAVCR2* axis by eLBP method. Meanwhile, we obtained 54 genes involved in cell communication. The 54-gene panel could also group the CCA patients into two subtypes, in which patients in S2 showed high expression level of immune related genes and had better prognosis. Finally, we constructed a nine-gene eLBP-LASSO-COX risk model which was designated as tumor microenvironment risk score (TMRS). The TMRS panel was revealed to be a reliable tool for prognostic prediction and chemotherapeutic decision-making in CCA. **(B)** Uniform manifold approximation and projection (UMAP) plot showing the cell composition in normal. GSEA enrichment plot showing the function of macrophage subtypes CD68_S100A9 and CD8 T cell subtype CD8_GZMA. NES: Normalized enrichment score. **(C)** UAMP plot showing the cell composition in cancer. GSEA enrichment plot showing the function of macrophage subtypes VEGFA_MACRO and CD8 T cell subtype TIGIT_CD8. NES: Normalized enrichment scores.

A schematic of the study design is shown in Figure 1A. To investigate the composition of the TME in CCA, we reanalyzed the single-cell data from GSE138709 (Zhang et al., 2020). We deconstructed the cell types in tumors and adjacent tissues. We observed evident differences between the two tissues. We obtained 16 cell types in the adjacent tissues, including CD8_GZMA, CD8_MKI67, and CD69-positive T cells, M1-like macrophage subtypes (CD68_C1QC, CD68_S100A9), and two DC subtypes (Figure 1B; Supplementary Figure S1A). GSEA showed that cytokine production and inflammatory responses were enriched in M1-like macrophage subtypes (CD68_C1QC and CD68_S100A9). Additionally, T cell activation and T cell-mediated immunity pathways were enriched in CD8_GZMA cells (Figure 1B). In tumor tissues, we obtained three T cell subtypes, including memory-like T cells (*CCR7*+ and CCR7_T) and exhausted CD8 (*TIGIT*+ and TIGIT_CD8). We also obtained M2-like macrophages (*VEGFA*+ and VEGFA_MACRO; Figure 1C; Supplementary Figure S1A). GSEA results showed that pathways including cell communication, cell motility, and response to stimulus were enriched in VEGFA_MACRO, while leukocyte activation and T cell differentiation were enriched in the TIGIT_CD8 subtype (Figure 1C). Interestingly, the sample distribution results showed that T cells and macrophages were mostly from samples 23T and 24T. In contrast, the cells from 18T to 20T samples were almost all epithelial cells (Methods; Supplementary Table S2). On this basis, the tumor samples in this dataset were divided into high- and low-TIL tumor subtypes. These results indicate that T cells and macrophages perform different functions in tumor and para-cancerous tissues.

### 3.2 Exploring the interacting cell type community and critical transcript regulons in tumor and paracancerous tissues using the eLBP algorithm

**FIGURE 2:**
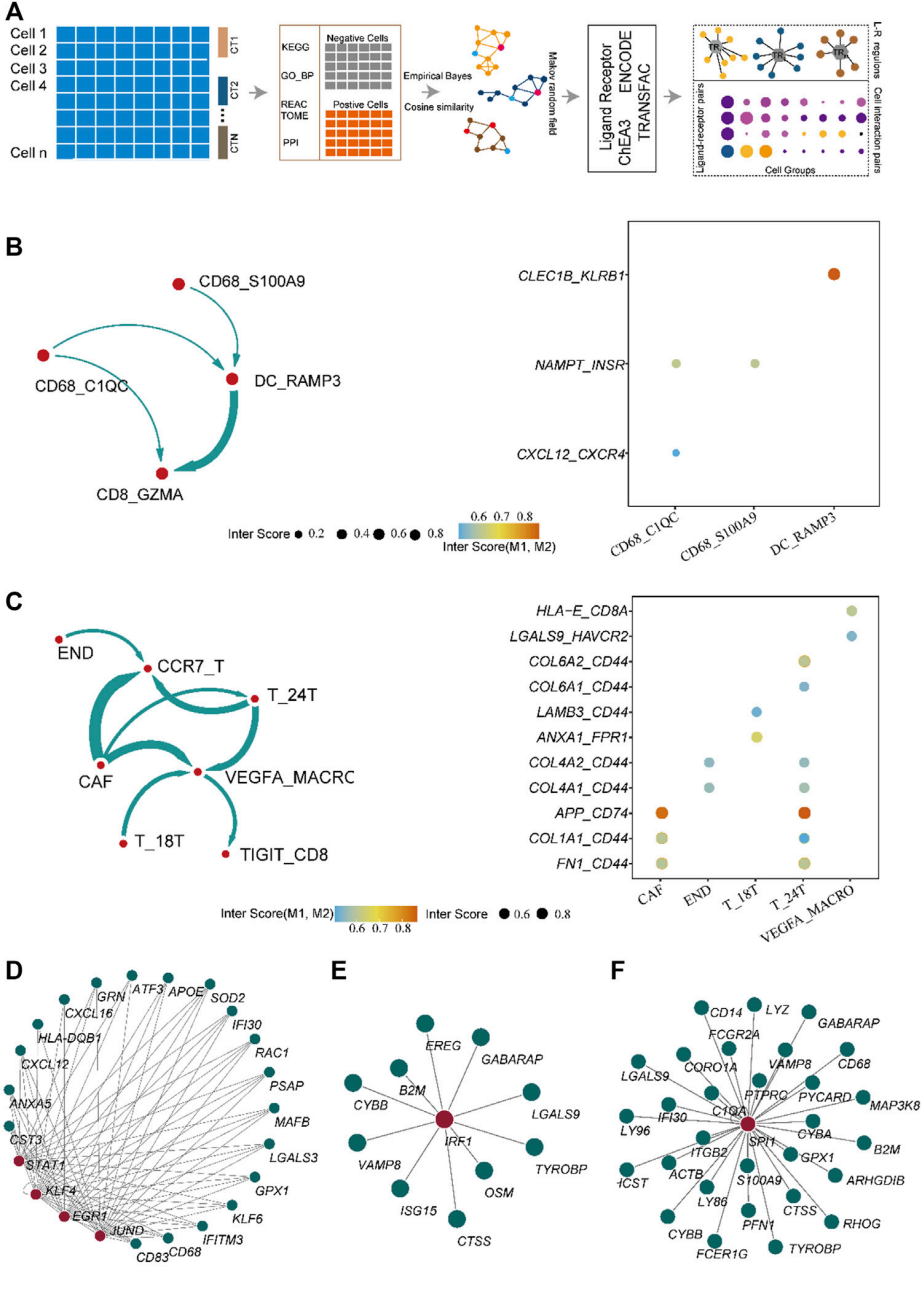
Algorithm framework of eLBP and the result calculated by eLBP. **(A)** Workflow of eLBP. Cell communication profile in the normal **(B)** (left) and cancer tissue **(C)**, in which line width was positive correlation with the cell interaction strength. Dot plot showing the interaction genes in the normal **(B)** and cancer tissue **(C)**, in which the row showing the interaction genes and the column showing the interaction cell types. The network showing the critical transcription factor (TF) regulons in normal **(D)** and cancer tissue **(E,F)**, in which red nodes were TFs and the other nodes were target genes.

To explore the mechanisms that induce the establishment of different immune states between normal and tumor tissues, we developed the eLBP algorithm to evaluate the correlation among immune-infiltrating, storm, and epithelial cells in normal and tumor single-cell data by calculating the intensity scores among the cell types (Figure 2A; Methods). First, we analyzed 7,071 activated cell type-specific pathways in normal tissues and 8,096 pathways in tumor tissues, in which 813 ligand or receptor genes in tumor tissues and 559 in normal tissues were computed based on the CellChat dataset (Supplementary Table S3). Second, we constructed protein-protein interaction (PPI) networks and calculated the cosine similarity between the PPI genes in each cell type. The degree of ligand/receptor (L/R) genes in the network is shown in Supplementary Figure S2A. The expression of ligand and receptor genes in CAF, CD68_C1QC, DC_RAMP3, and END was higher in normal tissues. T, 24T, TIGIT-CD8, and VEGFA_MACRO had higher degrees in the tumor tissues, indicating these cell types were in hyperactive status in cell communication among the TME (Supplementary Figure S2B). The levels of ligand and receptor genes indicate that macrophages with high levels of *VEGFA* play an important role in tumor tissues. Next, we used the eLBP algorithm to calculate the interaction probability of each ligand and receptor (Supplementary Table S3). Using this probability, we obtained the interaction intensity scores for each L/R pair among the cell types in the TME (Supplementary Tables S4, S5). The results demonstrated significantly different communities in tumors and normal tissues. In normal tissue, cells from DC_RAM3 and CD68_C1QC interacted with activated CD8 cells from CD8_GZMA according to NAMPT and chemokines, respectively (Figure 2B). In contrast, there were high levels of TIL infiltration in the tumor tissues. In addition, CAF could interact with memory-like T cells in CCR7_T, tumor cells in T_24T, and macrophages from VEGFA_MACRO. Moreover, VEGFA_MACRO interacted with exhausted CD8 + cells from TIGIT_CD8 (Figure 2C). The interaction genes between CAF and others are mainly related to cell adhesion, such as glycoprotein CD44 and collagen genes (Figure 2C). Meanwhile, VEGFA_MACRO, with a high level of LGALS9, interacted with *TIGIT* and *HAVCR2* (Figure 2C). These results illustrate that VEGFA_MACRO plays an important role in CD8 exhaustion.

**FIGURE 3:**
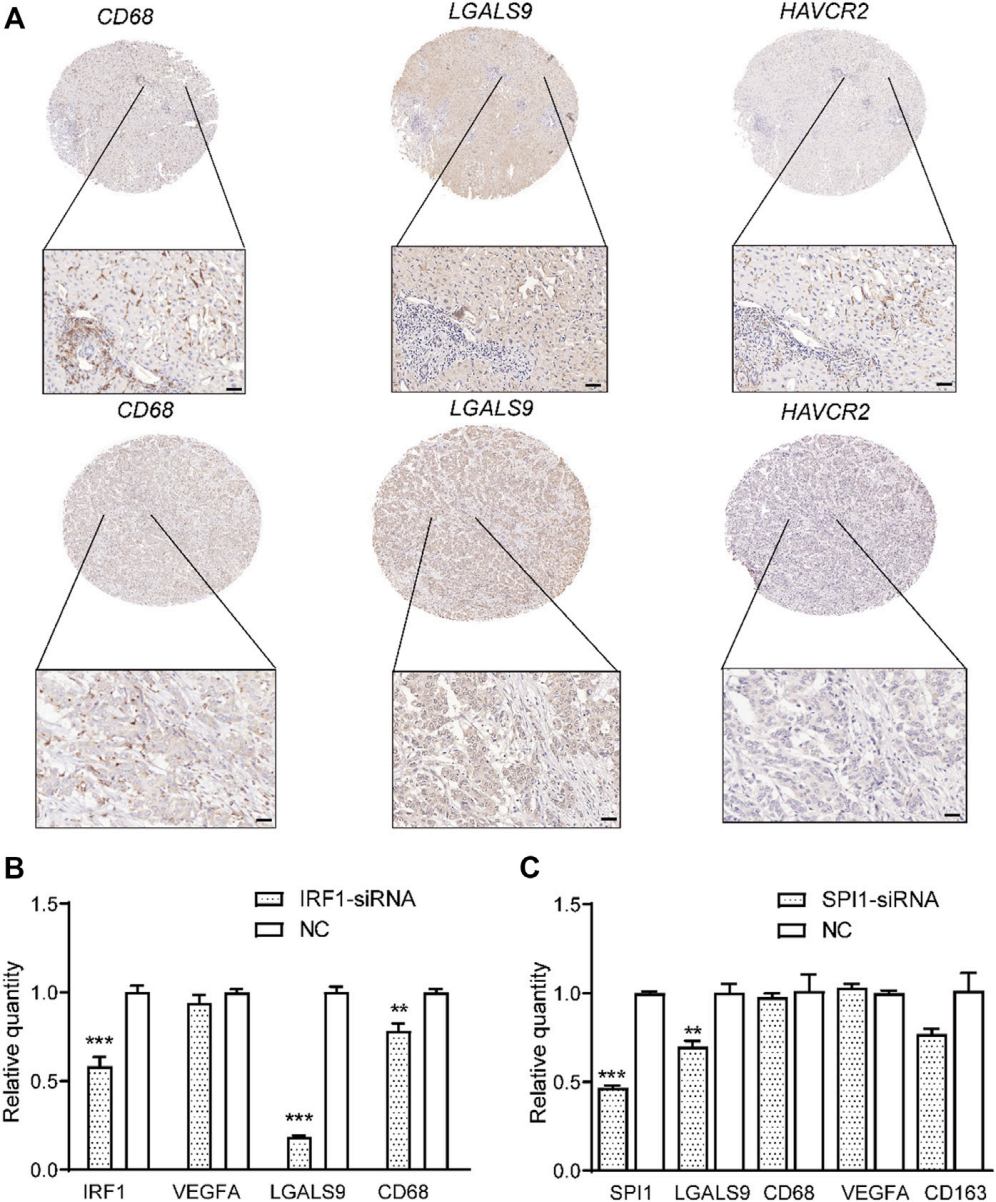
Validation of co-expression of *LGALS9* and *HAVCR2* and function of transcription factors *IRF1/SPI1*. **(A)** Immunohistochemical plot showing the co-expression of *LGALS9* and *HAVCR2* in two CCA patients. Scale bars represent 50 μm. Bar plot showing that TFs *IRF1* **(B)** and *SPI1* **(C)** could regulate the expression level of *LGALS9* (***p < 0.001; **p < 0.01, *t*-test).

Subsequently, we analyzed the key TFs that promote the expression of ligand genes in normal and cancerous tissues using the eLBP algorithm (Methods). In normal tissues, CD68_C1QC cells interacted with CD8_GZMA *via* CXCL12/CXCR4 (Figure 2B). We next obtained seven TFs (*STAT1*, *EGR1*, *KLF4*, *and JUND*; Figure 2D; Supplementary Table S6) from ENCODE and one TF (*KLF2*; Supplementary Table S6) from ChEA3. Moreover, *KLF4* and *STAT1* regulated the ligand gene *CXCL12* (Figure 2D), which may promote the expression of ligand genes and influence cell signaling. In addition, cells in DC_RAMP3 interact with those in CD8_GZMA *via CLEC1B/KLRB1* and TF *STAT3* in DC_RAMP3 could regulate *CLEC1B* (Supplementary Table S6). Notably, in the tumor tissues, we found that only the cells in VEGFA_MACRO interacted with those in TIGIT_CD8 *via LGALS9/HAVCR2* (Figure 2B). We then obtained 18 TF regulons from the three datasets, in which *IRF1* was enriched in both ENCODE and TRANSFAC, and *SPI1* was enriched in both ENCODE and ChEA3 (Supplementary Table S6). Moreover, these two TFs also regulated the ligand gene *LGALS9* (Figures 2E,F). In addition, the profiles of *LGALS9*, *HAVCR2*, *IRF1*, and *SPI1* in CCA patients from another single-cell dataset GSE151530 also verified their functions related to TAM and exhausted T cells (Supplementary Figure S3) (Ma et al., 2021). Moreover, immunohistochemical analysis further confirmed the high expression of *HAVCR2* in human CCA patients and the co-expression of *LGALS9* and these two genes (Figure 3A). The significantly low expression of *LGALS9* in siRNA-IRF1/SPI1 VEGFA-positive macrophages further validated the transcript regulon of SPI1/IRF1-LGALS9 (Figures 3B,C). Finally, we derived the gene trimmed-PPI network from the mutually connected cell types in which L/R genes participate in normal and tumor tissues *via* eLBP (Supplementary Figure S4).

### 3.3 Cell communication genes obtained from eLBP can be used to distinguish inflammatory subtype from a replicated one

**FIGURE 4:**
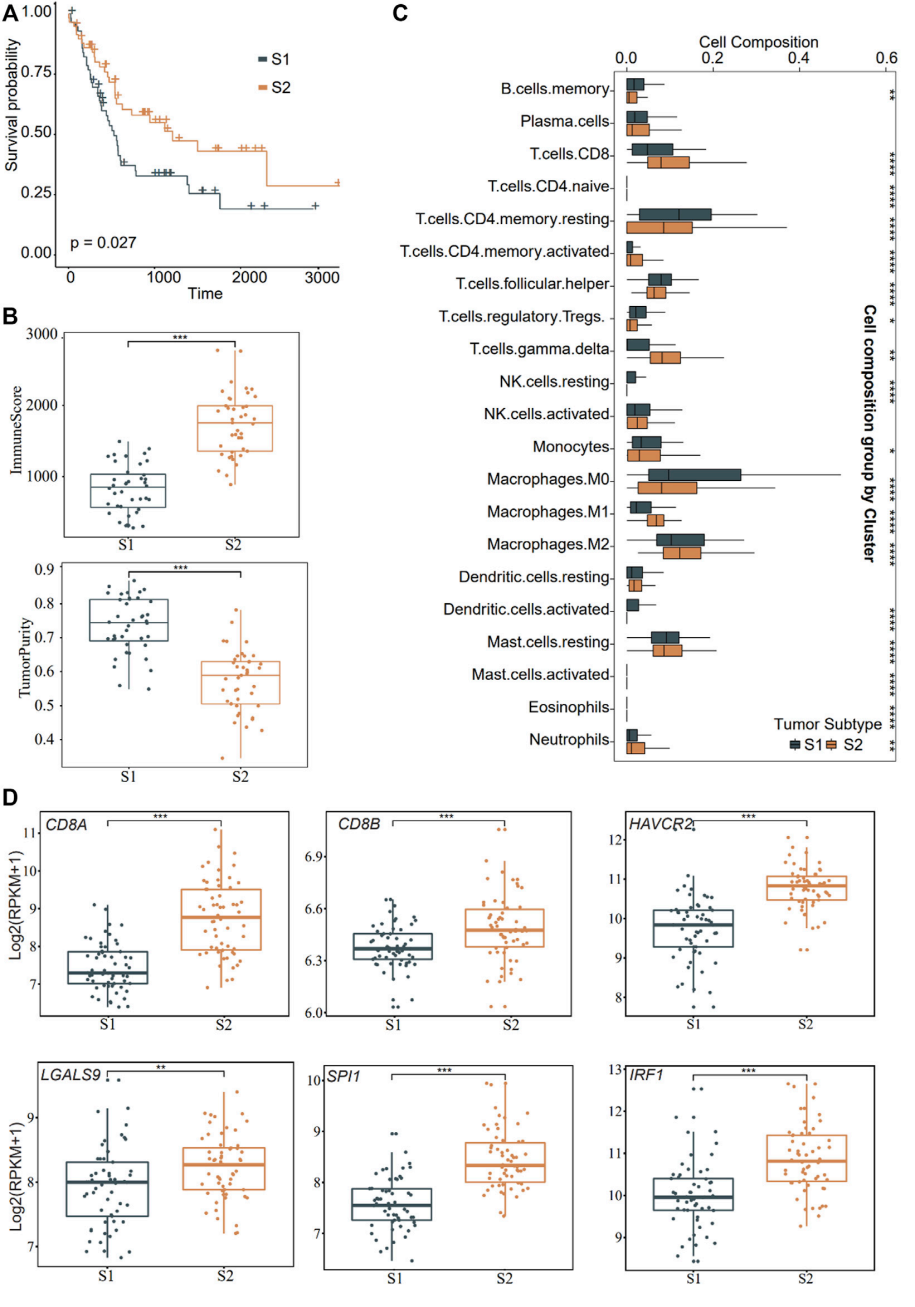
Distinct function in the two subtypes of CCA. **(A)** Overall survival curves showing the prognosis result of the two subtypes (S1 and S2) obtained from consensus clustering in CCA cohort (GSE89749) using the genes obtained from eLBP. Statistical significance was calculated using the log-rank test. **(B)** Box plots showing the immune and tumor purity scores in the two distinct malignant subtypes (***p < 0.001). Pairwise comparison was conducted by Wilcoxon rank sum test. **(C)** Box plots showing the 22 immune cell infiltrates ratio in the two distinct malignant subtypes in the significant enrich patients. (*p < 0.05; **p < 0.01; ***p < 0.001; Wilcoxon rank sum test). **(D)** Comparisons of gene expression level of immune genes in the two distinct malignant subtypes (**p < 0.01; ***p < 0.001; Wilcoxon rank sum test). For the boxplot, the centerline, median; box limits, upper and lower quartiles. Each dot represents a sample.

Concerning the potential function of VEGFA_MACRO to induce the expression of exhausted genes such as *HAVCR2*, we further overlapped the genes in TF regulons with those in ligand or receptor-interaction trimmed-networks from eLBP from VEGFA_MACRO and obtained 54 genes (Supplementary Figure S4C; Supplementary Table S7). We then investigated the clinical features of patients with CCA using these genes. We grouped patients into different subtypes in two bulk RNA datasets using consistent clustering (Methods; GSE89749; GSE76297; Supplementary Table S1) (Chaisaingmongkol et al., 2017; Jusakul et al., 2017). Patients in GSE89749 were grouped into two subtypes (S1 and S2; Figure 4A; Supplementary Figure S5). Moreover, we found that the samples from the S2 group had a significantly better prognosis (*p* = 0.027; Figure 4A). Notably, the tumor purity results showed that patients in S2 had higher infiltrative immune and stromal scores, but lower tumor purity (Figure 4B). The immune infiltration results showed that patients in S2 had a higher ratio of CD8, activated memory CD4, S1, and M2-like macrophage subtypes (Figure 4C). In particular, *CD8A*, *CD8B*, *CD68*, *HAVCR2*, and *LGALS9* were highly expressed in S2 (Figure 4D). On this basis, S2 was classified as the inflammatory “hot tumor” subtype and S1 as the “cold tumor”. We further identified the clustering results from other RNA sequencing datasets (GSE76297). The patients in S2 from GSE76297 had higher expression levels of *LGALS9*, *IRF1*, *SPI1*, and immune genes and similar TIL profiles with the GSE89749 data (Supplementary Figure S6). These results further confirmed the existence of high-TIL tumor subtypes in CCA and indicated the exhaustion of CD8 cells.

### 3.4 Inter-tumor heterogeneity between inflammatory and replicated subtypes

**FIGURE 5:**
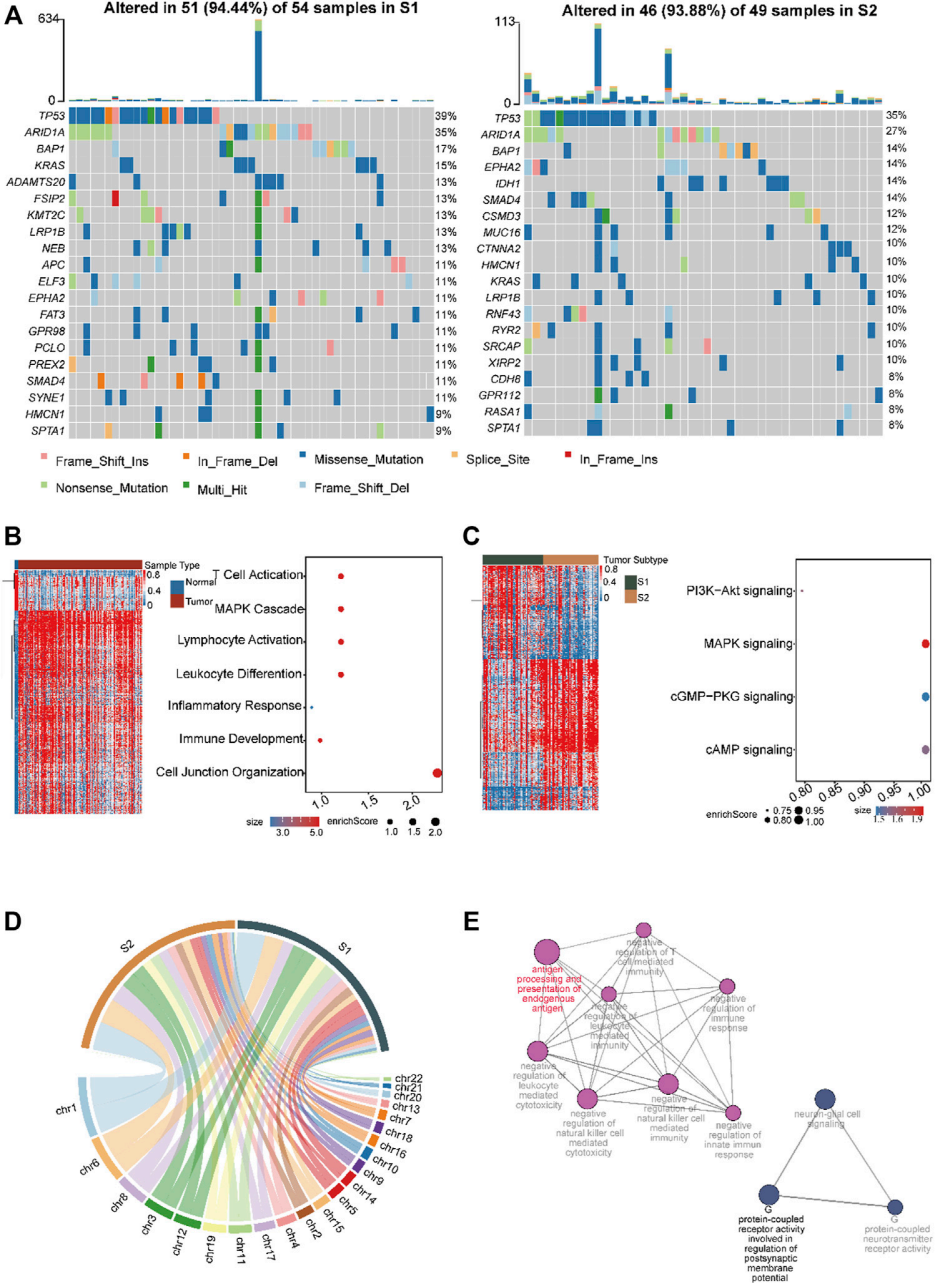
Investigation of the mutation profile inter-tumor heterogeneity profiles in the two CCA subtypes. **(A)** The top ten mutated genes across the two subtypes. The colors of rectangles in the body of the heat map indicate different types of somatic mutations and the key identifying each mutation type is shown at the bottom below the color bar. The bar plot on the top shows the counts of mutations for each patient and the colors in the bar plots correspond to the colors showing mutation types in the body of the heat map. The title of the heat map showing the mutation sample number in each subtype including amplification, missense mutations, and deep deletions. The left number showing the gene mutation frequency in the two subtypes. Heatmap and dot plot showing the differentially expressed genes and the significantly enriched pathways in normal **(B)** and cancer tissue **(C)** in CCA. Color gradient blue to red indicates relative expression levels from low to high. Dot size represent enrichment scores. **(D)** Circos plot showing the amplification and deletion regions in the two subtypes. The width of the plot indicated the CNV mutation regions numbers in each subtype across the chromosomes. **(E)** Network showing enriched pathways of hypo-methylated genes that occur on chromosomes 6 and 8 in S2. Dot size represents the enrichment scores.

Next, we analyzed the heterogeneity of somatic frequencies across the two CCA subtypes. The results showed that the cell replication genes TP53 and genes in the PI3K/Akt pathway, such as *BAP1*, *KRAS*, *EPHA2*, *ARID1A*, and *SMAD4*, exhibited high mutation ratios in the two subtypes (Figure 4A). S1 had high ratios of *ADAMTS20*, *KMT2C*, and *APC* (Figure 5A, 13%, 13%, and 11%, respectively). S2 had a higher mutation frequency of *IDH1* and *MUC16* (Figure 5A, 14% and 12%, respectively). Tumor cells with TP53 mutations are generally identified as being more immunogenic (Chasov et al., 2020), and we found that immune genes, such as *CD3D* and *HAVCR2*, were highly expressed in the *MUC16* mutated group. Subsequently, we investigated the heterogeneity of methylation profiles between normal tissues and tumors. We obtained 15,520 differential probes between normal and tumor tissues, including 5,219 downregulated and 10, 321 upregulated probes (Methods, Figure 5B). In normal tissues, hypomethylated genes were mainly enriched in pathways such as T cell differentiation, cell adhesion, and inflammatory response, which were also enriched in normal single-cell datasets (Figure 5B). This indicated a high immune infiltration and activated immune status of the normal. Among the two CCA subtypes, we obtained 697 downregulated probes and 1,115 upregulated probes in S2. In addition, there were 684 downregulated hypomethylated probes enriched in 271 genes in S2. These genes were enriched in MAPK, CAMP, cGMP-PKG, and PI3K-AKT pathways (Figure 5C). We then conducted CNV analysis using the methylation data. The results showed that significant CNV signaling occurred across chromosomes 1, 6, and 8 in both subtypes (Figure 5D). Interestingly, we found that S2 had more CNV regions on chromosomes 6 and 8 (Figure 5D). We then analyzed the hypomethylated genes on chromosomes 6 and 8 in S2. These genes were enriched in antigen presentation and immune-toxic suppression, as well as T cell differentiation and G protein-coupled receptor-associated signaling pathways (Figure 5E).

### 3.5 Construct an eLBP-COX risk model to distinguish between inflammatory and replicated subtypes

**FIGURE 6:**
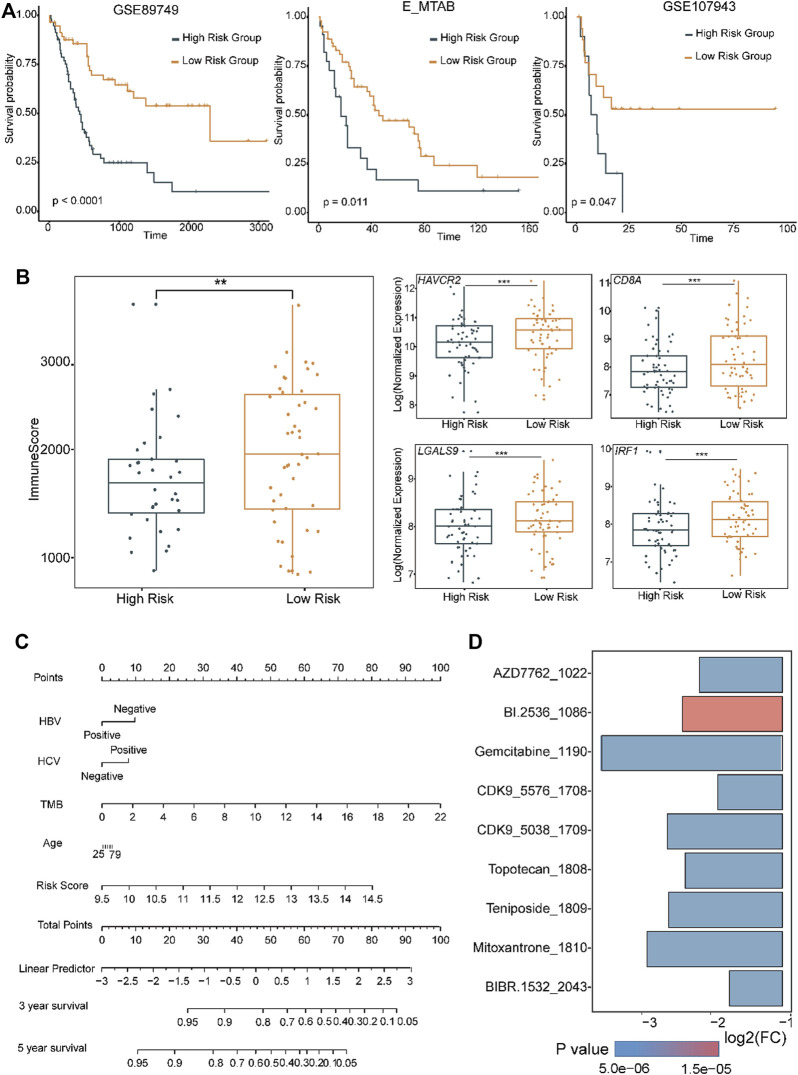
Distinct functions in different level of tumor microenvironment risk scores. **(A)** Overall survival curves showing the prognosis results with different level of tumor microenvironment risk score (TMRS) in the two CCA cohorts using the genes obtained from eLBP-LASSO. Statistical significance was calculated using the log-rank test. **(B)** Box plots showing the immune and the exhausted related genes in high and low risk group (**p < 0.01; ***p < 0.001; Wilcoxon rank sum test). **(C)** Nomogram plot showing the predicted 3- and 5-year survival possibilities of individual CCA patients. **(D)** Bar plot showing the drug sensitivity in S2 subtype.

LASSO-COX regression was used to construct the eLBP-COX TME risk score (TMRS) using the following formula in dataset GSE89749: TMRS = −0.03085 × *IRF1* − 1.03491 × *RHOA* + 0.5868 × *PLAUR* − 0.2188 × *NCF2* − 0.2273×*CXCR4* − 0.1201 × *HCK* − 0.1773 × *LYZ* − 0.1244 × *RGS1* + 0.2259 × *VCAN* (Methods; Supplementary Figure S7). We found that patients with low TMRS had a better prognosis with higher immune scores and presented with *IRF1, LGALS9*, and other immune genes in GSE89749 (Figures 6A,B), indicating an effective indicator of RS score in prognostic risk prediction. Furthermore, we verified the RS model in two other datasets (EMTAB-6389; GSE107943 Supplementary Table S1; Methods; Figure 6A) (Ahn et al., 2019). Meanwhile, a nomogram was drawn to visualize the results of the eLBP-COX regression analysis (Figure 6C). Subsequently, we conducted drug susceptibility analysis and explored seven drugs (AZD7762, BI 2536, CDK9 5576, gemcitabine, mitoxantrone, teniposide, and topotecan) with IC50 values that were significantly lower in S2 than in S1 (Methods; Figure 6D). Specifically, BI 2536, teniposide, and mitoxantrone were used as potential therapeutic drugs for HCC that could be used in the treatment of CCA.

## 4 Discussion

Most patients with CCA are usually diagnosed at advanced stages and are not eligible for curative resection, with an overall survival (OS) of less than 1 year from the time of diagnosis. In the last decade, several novel therapeutic interventions and agents have improved the clinical outcomes of patients with CCAs. For example, radiofrequency ablation and mFOLFOX plus active symptom control (ASC) could significantly improve OS after progression on cisplatin-gemcitabine combination therapy compared to ASC alone (Rizzo et al., 2020). Multi-omics studies have revealed that FGFR2 fusions and rearrangements, IDH-1 mutations, and BRAF mutations are frequently found in patients with CCA. Targeted therapies such as the FGFR2 inhibitor pemigatinib and IDH1 inhibitor ivosidenib potentiate the treatment of CCAs harboring these mutations (Brandi et al., 2020). However, polyclonal mutations can also induce resistance to target drugs. Novel therapeutic strategies, such as immunotherapy, are needed, and immunotherapy is emerging as an important approach to cancer treatment. However, its efficacy varies greatly among cancer patients (Murciano-Goroff et al., 2020). CCA possesses a special TME that can be classified as either immune “hot” or “cold”. Of these, immune “hot” tumors have a better response to immunotherapies (Loeuillard et al., 2019). ICIs represent a revolutionary milestone in the field of immuno-oncology (Darvin et al., 2018). The most common ICIs currently in clinical trials are PD-1/PD-L1 and *CTLA4* (Rizzo et al., 2021b)*.* The response rates to ICIs vary owing to the complex composition of the TME (Yao and Gong, 2021). Therefore, we systematically investigated the composition of the TME to explore the critical interactions between cell types and signal transduction in CCAs.

First, we analyzed the TME composition of patients with CCAs using single-cell sequencing data. We found that activated CD8 cells and M1-like macrophages infiltrated normal tissues but exhausted CD8 cells in tumors. We further explored the critical elements in the signaling transduction pathways among CCA TMEs, such as the most frequently interacting cell types, the corresponding activated interaction gene pairs, the key transcription factors, and the gene regulons, by integrating L/R interactions with intracellular signaling *via* the Markov Random Fields model and developed an eLBP algorithm. We adopted the algorithm and found that macrophages with high expression levels of *LGALS9* could interact with exhausted CD8 T cells expressing high levels of *HAVCR2* in tumor tissues in CCAs. It is reported that galectin-9 (*LGALS9*) could interact with PD-1 and TIM-3 (*HAVCR2*) to regulate T cell death in multi-cell lines (Yang et al., 2021). Meanwhile, we first found that TFs, such as *IRF1* and *SPI1*, may promote the expression of *LGALS9* and verified their expression through multiple datasets and immunohistochemical staining in CCA tissues. It has been reported to *IRF1* inhibits antitumor immunity through upregulation of PD-L1 in MC38 and CT26 colon cancer and B16 melanoma mouse models (Shao et al., 2019). It has been demonstrated that *Spi1* is required for myeloid-specific expression of *Lgals9* in zebrafish (Zakrzewska et al., 2010). However, their function in CCAs merits further investigation.

Second, eLBP provided a gene classifier consisting of 54 genes that dominated the signal transduction between macrophages and exhausted CD8 T cells. This classifier can group CCA patients into replicated subtypes S1 and S2. Interestingly, patients with the inflammatory subtype S2 with high levels of *LGALS9* and *HAVCR2* had a better prognosis. Meanwhile, a higher mutation ratio of *MUC16* in S2 was positive for immune-exhausted genes, such as *HAVCR2* and *TIGIT*. Recent studies have demonstrated that *MUC16* mutations are associated with better survival outcomes and immune responses in gastric and endometrial cancers (Wang et al., 2020).

Pathological confirmation of the diagnosis is necessary before any nonsurgical treatment but is challenging in CCA therapy. Endoscopic imaging and tissue sampling are useful; however, biopsy samples are often inadequate for molecular profiling. Tissue sampling is reported to be highly specific but has low sensitivity in the diagnosis of malignant biliary strictures. Liquid biopsy can capture and monitor the tumor genetic profile or response to therapy in real time. For example, high mutation rates of AR1D1A, FGFR2, PIK3CA, and TP53 proteins, high levels of microRNAs such as miR-21, and high levels of proteins and cytokines such as CK-19, MMP-7, osteopontin, periostin, and IL-6 have been found in CCA patient serum (Rompianesi, et al., 2021). These protein and microRNA markers can serve as prognostic and therapeutic markers. In this study, we set up a 54-gene panel to screen or predict patients that are suitable for immunotherapy and finally constructed a nine-gene eLBP-LASSO-COX risk model. From this model, we found that *IRF1* was positively associated with low risk, while *PLAUR* and *VCAN* were positively associated with a high-risk score in tumorigenesis of CCAs. It has been reported that *PLAUR* overexpression correlates with poor prognosis in HCC (Woo et al., 2008). Glioma patients with higher *PLAUR* expression are infiltrated with fewer CD8 T cells (Zeng et al., 2021). *VCAN* has been used as an immune exclusion marker (Emmerich et al., 2020). To facilitate clinical application, we will further investigate their expression levels in serum and exosomes and their capacity for the early screening of CCAs.

## 5 Permission to reuse and copyright

Figures, tables, and images will be published under a Creative Commons CC-BY licence and permission must be obtained for use of copyrighted material from other sources (including re-published/adapted/modified/partial figures and images from the internet). It is the responsibility of the authors to acquire the licenses, to follow any citation instructions requested by third-party rights holders, and cover any supplementary charges.

## 6 Nomenclature

### 6.1 Resource identification initiative

To take part in the Resource Identification Initiative, the corresponding catalogue numbers were listed in Table 1.

**TABLE 1:** Resource identification initiative information.

Name	Categorization	Supplier	Cat no.
CD68	Protein	CST	76437T
LGALS9	Protein	Abcam	ab153673
HAVCR2	Protein	Abcam	ab153673

## Data Availability

The original contributions presented in the study are included in the article/Supplementary Material, further inquiries can be directed to the corresponding author.

## Abbreviations

CAF, cancer-associated fibroblasts; CCA, cholangiocarcinoma; CP1, checkpoint inhibitors; DC, dendritic cells; DEGs, differential genes; eLBP, empirical Bayes loopy belief propagation; iCCA, intrahepatic; ICIs, immune checkpoint inhibitors; ICGs, mimmune checkpoint genes; LASSO, least absolute shrinkage and selection operator; MRF, Markov random field; TF, transcription factors; TMRS, tumor microenvironment risk score; TMEs, Tumor microenvironments; UMAP, uniform manifold approximation and projection.
