# Supplementary material for: Integrating cell interaction with transcription factors to obtain a robust gene panel for prognostic prediction and therapies in cholangiocarcinoma
Source: Front Genet. 2022 Nov 30;13:981145. doi: 10.3389/fgene.2022.981145 (PMC9748417; doi:10.3389/fgene.2022.981145)
Supplement: Supplementary file 1 [file DataSheet1.pdf]

Integrating cell interaction with transcript factors to obtain a robust gene panel for prognostic prediction and therapies in cholangiocarcinoma

Tingjie Wang, Chuanrui Xu, Dan Xu, Xiaofei Yang , Yaxin Liu, Xiujuan Li,

Zihang Li, Ningxin Dang, Yi Lv, Zhijing Zhang, Lei Li and Kai Ye

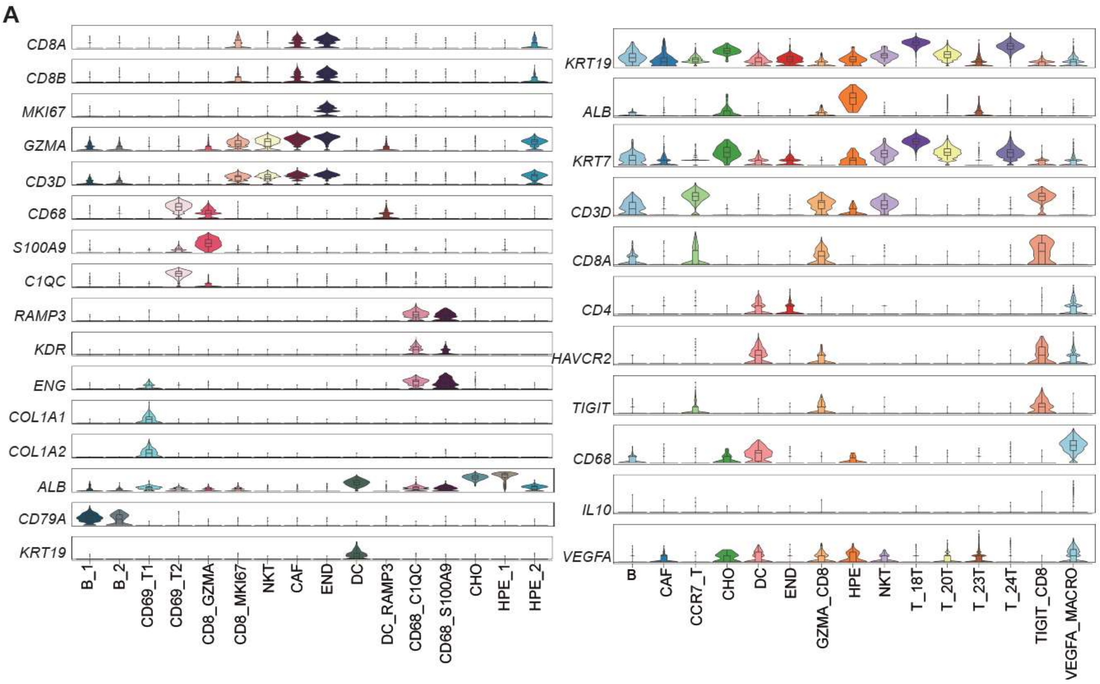

Figure S1: Gene profile in normal and cancer in CCA single cell RNAseq dataset (GSE138709).

(A) Violin plots showing marker genes for four distinct endothelial and five fibroblast subtypes, colored according to cell types.

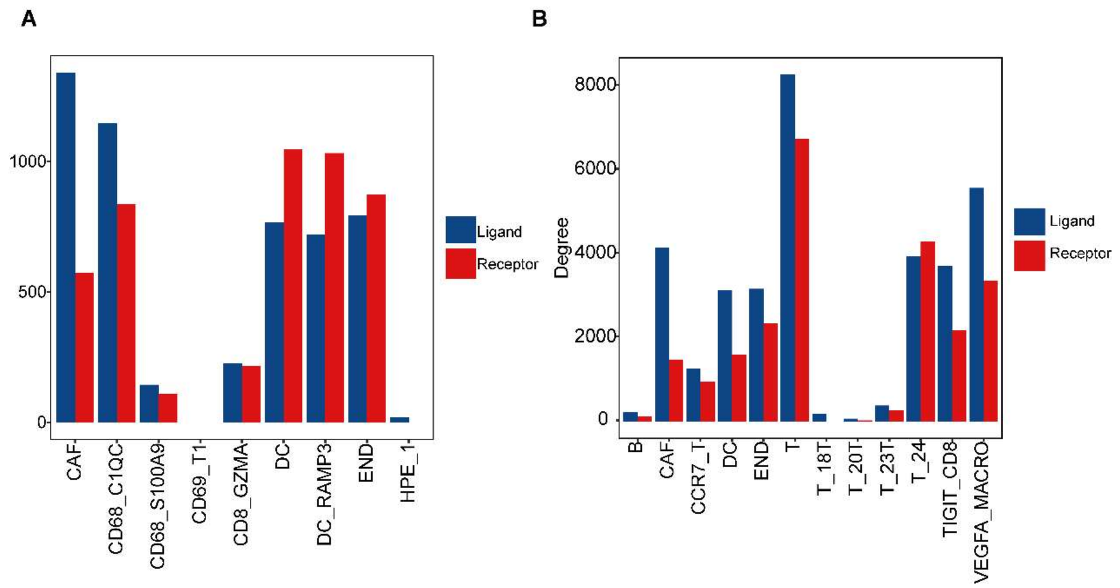

Figure S2: Cell interaction genes in normal and cancer in CCA single cell RNAseq dataset (GSE138709).

Ligand (blue) and receptor (red) gene numbers in normal (A) and cancer (B) in CCA single cell RNAseq dataset (GSE138709).

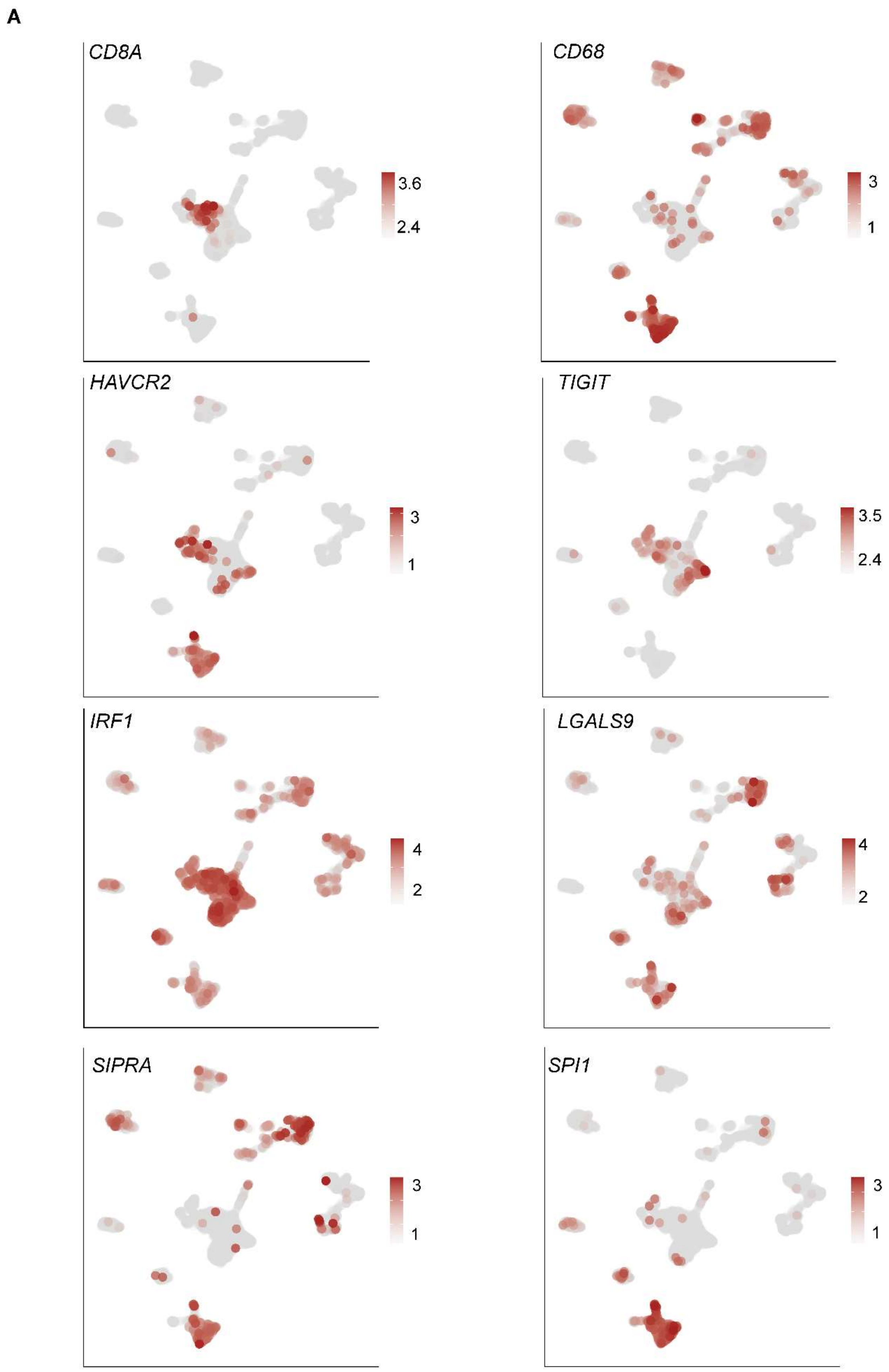

Figure S3: Validation the critical genes in CCA single cell RNAseq dataset

(GSE151530).

UMAP plot showing the expression level of marker genes in the other single cell dataset GSE151530. Color from grey to red represent the expression level from low to high.

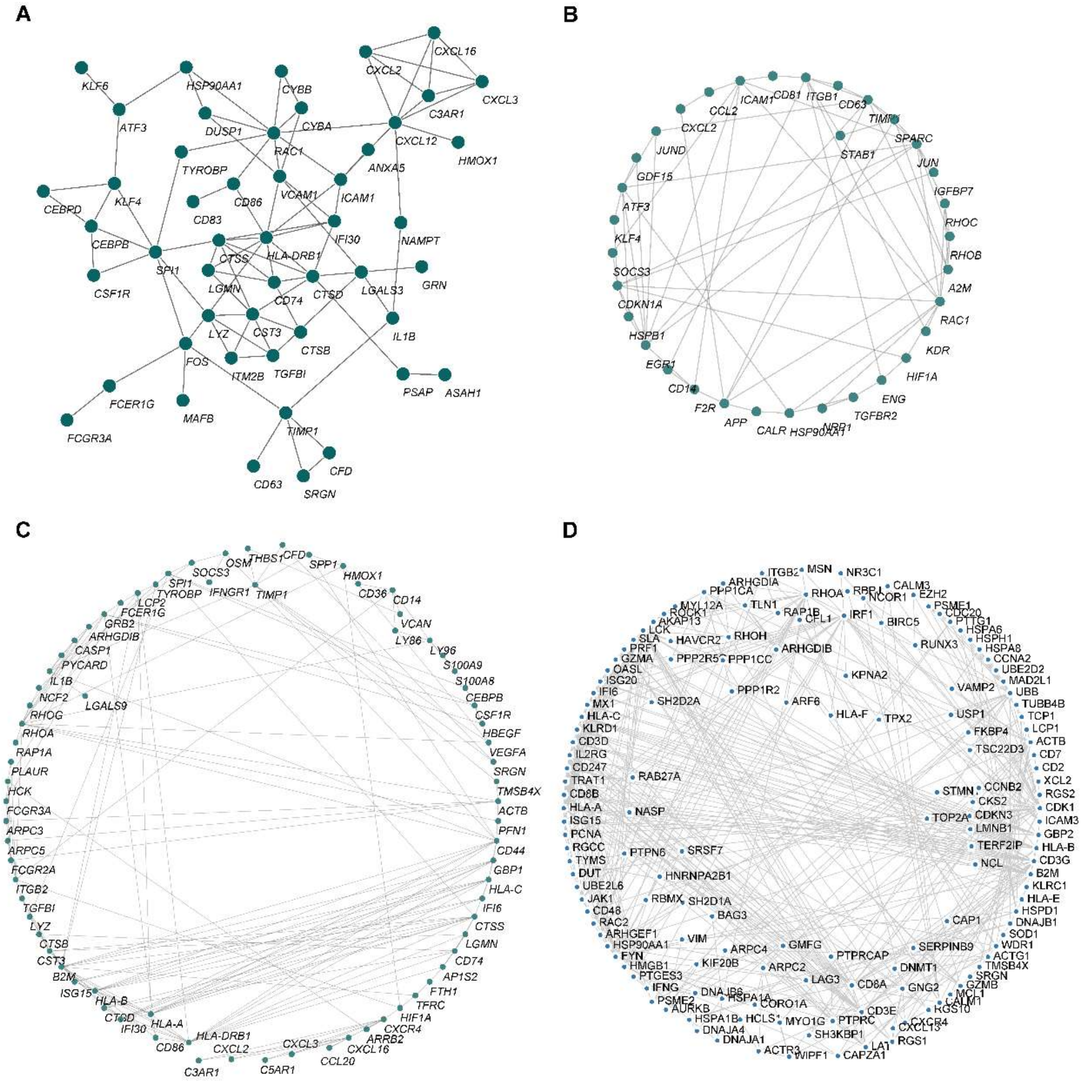

Figure S4: Trimmed-PPI network from the mutually connected cell types that ligand or receptor genes participate in normal and tumor tissues via eLBP.

(A) PPI network that ligand gene CXCL12 participated in the cell type CD68_C1QC from normal tissue. (B) PPI network that ligand gene CLEC1B

participated in the cell type DC_RAMP3 from normal tissue. (C) PPI network that ligand gene LGALS9 participated in the cell type VEGFA_MACRO. (D) PPI network that receptor gene HAVCR2 participated in the cell type TIGIT_CD8.

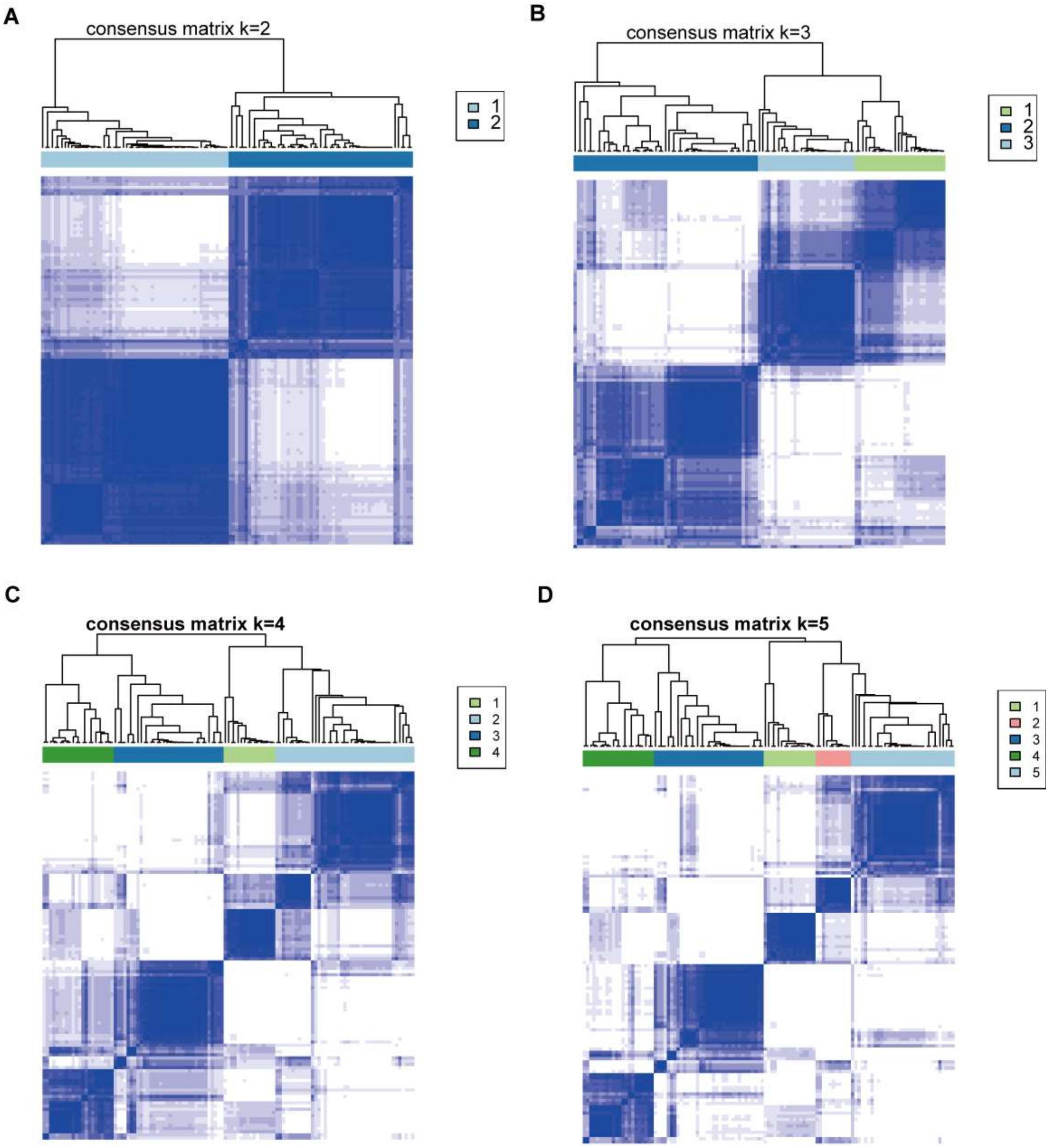

Figure S5: Heatmap showing the consensus cluster result using different cluster number.

Heatmap showing the consensus cluster result using different cluster number from 2 to 5 (A-D).

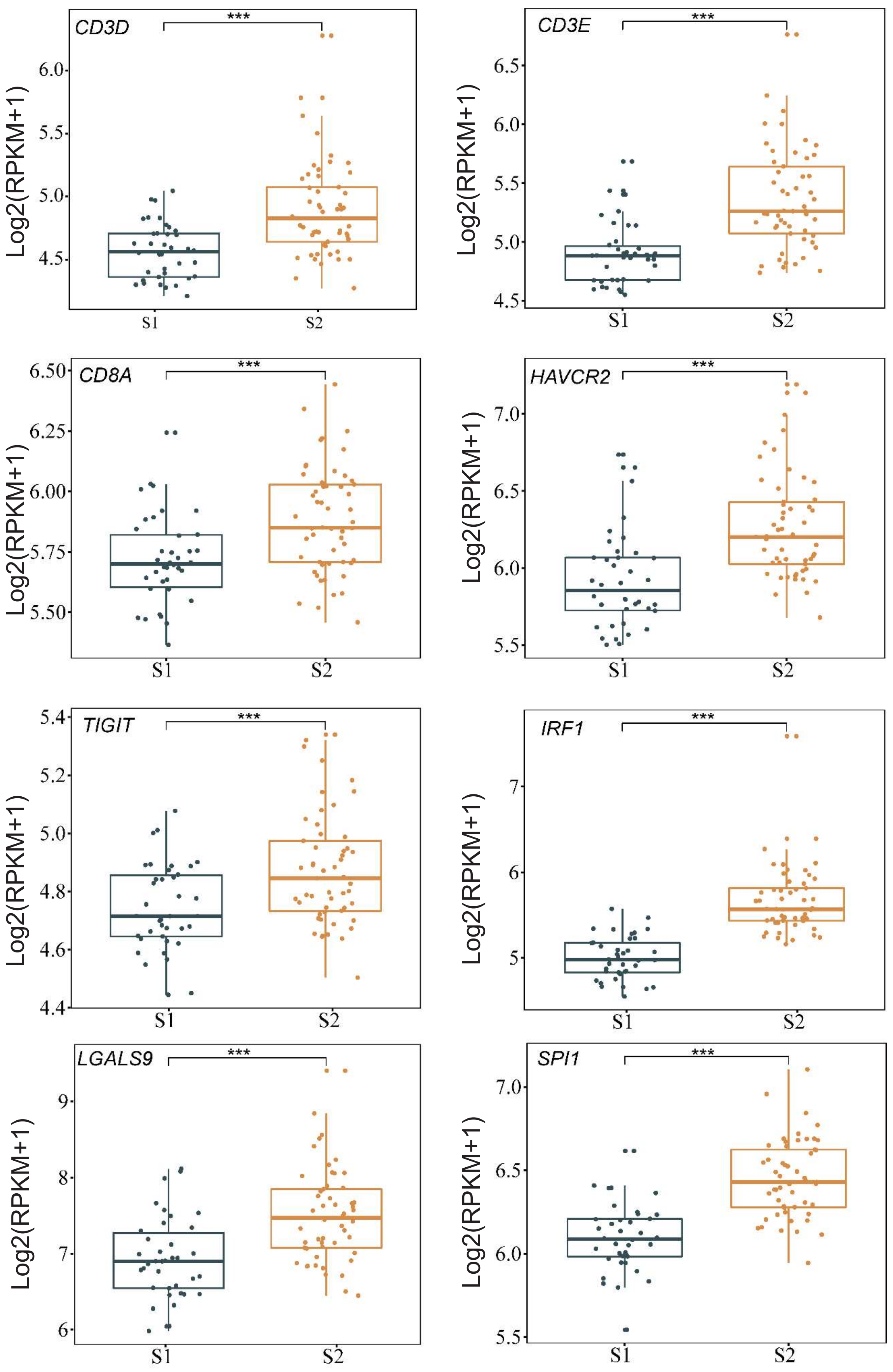

Figure S6: Validation the gene expression level of critical genes in CCA cohort

GSE76297.

Box plot showing the patients in S2 from GSE76297 had higher expression levels of *LGALS9, IRF1, SPI1* and immune genes, and similar TIL profiles with the GSE89749 data (***P < 0.001, Wilcoxon rank sum test).

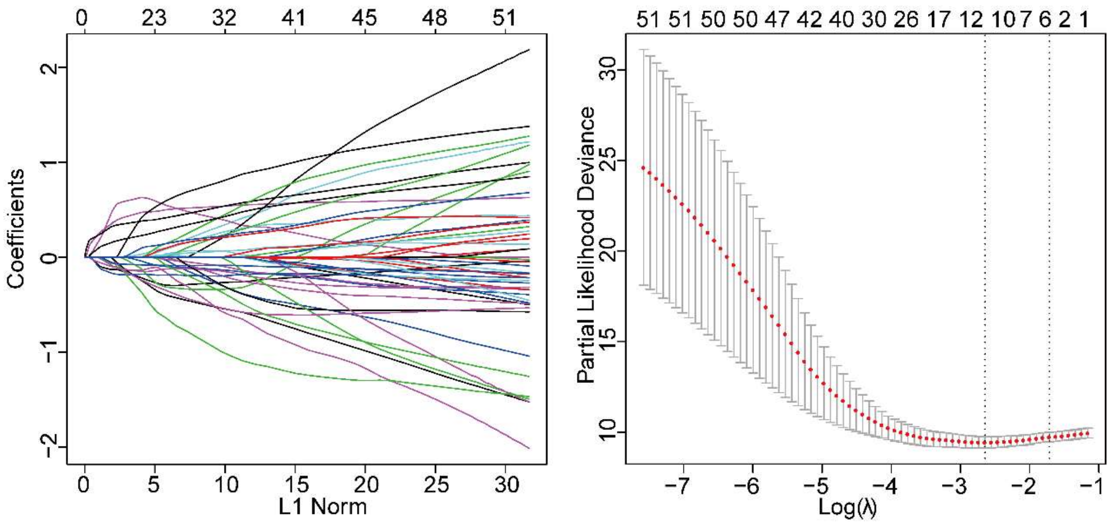

Figure S7: LASSO regression plot showing the signatures selection process.

(A) Lambda value of the 52 genes in least absolute shrinkage and selection operator (LASSO) model. (B) The most appropriate log (Lambda) value in the LASSO model.

Table S1. Deposited data.

Dataset	Type	Site	patient number	prognosis
GSE138709	single cell	https://www.ncbi.nlm.nih.gov/geo/query/acc.cgi?acc=GSE138709	Adjacent:3, Tumor:5	No
GSE151530	single cell	https://www.ncbi.nlm.nih.gov/geo/query/acc.cgi?acc=GSE151530	Tumor:12	No
GSE89749	Bulk	https://www.ncbi.nlm.nih.gov/geo/query/acc.cgi?acc=GSE89749	Tumor:138;Adjacent:4	Yes
GSE107943	Bulk	https://www.ncbi.nlm.nih.gov/geo/query/acc.cgi?acc=GSE107943	Tumor:30;Adjacent:27	Yes
GSE76297	Bulk	https://www.ncbi.nlm.nih.gov/geo/query/acc.cgi?acc=GSE76297	Tumor:91;Adjacent:92	No
E-MTAB-6389	Bulk	https://www.ebi.ac.uk/arrayexpress/experiments/E-MTAB-6389/	Tumor:78;Adjacent:31	Yes

Table S2. Cell type distribution between normal and tumor tissues.

Patients	Cell Type	Enrich Scores	Patients	Cell Type	Enrich Scores
18T	B	0.03	23T	HPE	0.49
20T	B	0.12	24T	HPE	2.54
23T	B	0.26	18T	NKT	0
24T	B	2.66	20T	NKT	6.07
18T	CAF	0.13	23T	NKT	0
20T	CAF	0.17	24T	NKT	0.11
23T	CAF	0.49	18T	T_18T	3.44
24T	CAF	2.41	20T	T_18T	0
18T	CCR7_T	0.02	23T	T_18T	0
20T	CCR7_T	1.15	24T	T_18T	0
23T	CCR7_T	0.31	18T	T_20T	0
24T	CCR7_T	2.16	20T	T_20T	6.27
18T	CHO	0	23T	T_20T	0
20T	CHO	0	24T	T_20T	0.01
23T	CHO	0.41	18T	T_23T	0
24T	CHO	2.65	20T	T_23T	0
18T	DC	0.39	23T	T_23T	4.88
20T	DC	1.03	24T	T_23T	0
23T	DC	0.3	18T	T_24T	0.01
24T	DC	1.92	20T	T_24T	0
18T	END	0.08	23T	T_24T	0
20T	END	0.33	24T	T_24T	2.88
23T	END	0.32	18T	TIGIT_CD8	0.04
24T	END	2.49	20T	TIGIT_CD8	0.13
18T	GZMA_CD8	0	23T	TIGIT_CD8	3.74
20T	GZMA_CD8	0	24T	TIGIT_CD8	0.58
23T	GZMA_CD8	4.88	18T	VEGFA_MACRO	0.21
24T	GZMA_CD8	0	20T	VEGFA_MACRO	0.28
18T	HPE	0	23T	VEGFA_MACRO	1.18
20T	HPE	0.13	24T	VEGFA_MACRO	1.89

Table S3. Scores of ligand or receptor genes participated in the cell communication calculated by eLBP algorithm.

CellType	Gene	L/R value	Type	Sample Type
B	CD27	0.937418	receptor	Tumor
B	CXCR4	0.872329	receptor	Tumor
B	HLA-A	0.568182	ligand	Tumor
B	HLA-B	0.60824	ligand	Tumor
B	HLA-C	0.954512	ligand	Tumor
B	ICAM2	0.975225	receptor	Tumor
B	IFNAR2	0.304385	receptor	Tumor
B	IL2RG	0.808196	receptor	Tumor
CAF	ANGPT2	0.007568	ligand	Tumor
CAF	ANGPTL4	0.017046	ligand	Tumor
CAF	APP	0.965958	ligand	Tumor
CAF	C3	0.131565	ligand	Tumor
CAF	CCL2	0.245411	ligand	Tumor
CAF	COL1A1	0.999285	ligand	Tumor
CAF	COL1A2	0.026041	ligand	Tumor
CAF	COL4A1	0.460353	ligand	Tumor
CAF	COL4A2	0.402108	ligand	Tumor
CAF	COL6A1	0.434307	ligand	Tumor
CAF	COL6A2	0.630021	ligand	Tumor
CAF	COL6A3	0.072348	ligand	Tumor
CAF	FN1	0.99785	ligand	Tumor
CAF	IL6	0.998801	ligand	Tumor
CAF	ITGA1	0.001653	receptor	Tumor
CAF	LRP1	0.123011	receptor	Tumor
CAF	MCAM	0.313007	receptor	Tumor
CAF	NOTCH3	0.999999	receptor	Tumor
CAF	PDGFRB	0.04058	receptor	Tumor
CAF	PGF	0.021136	ligand	Tumor
CAF	POSTN	0.038931	ligand	Tumor
CAF	SDC2	0.028548	receptor	Tumor
CAF	THBS1	0.972057	ligand	Tumor
CAF	THBS2	0.066542	ligand	Tumor
CAF	THY1	0.000223	ligand	Tumor
CCR7_T	CCR6	0.007494	receptor	Tumor
CCR7_T	CCR7	0.426381	receptor	Tumor
CCR7_T	CD44	0.773747	receptor	Tumor
CCR7_T	CXCR6	0.796945	receptor	Tumor

CCR7_T	GZMA	0.999861	ligand	Tumor
CCR7_T	IL2RG	0.987716	receptor	Tumor
CCR7_T	IL7R	0.502258	receptor	Tumor
CCR7_T	ITGB2	0.828232	receptor	Tumor
CCR7_T	TGFB1	0.999864	ligand	Tumor
CCR7_T	TNFRSF1B	0.659264	receptor	Tumor
CCR7_T	TNFRSF4	0.355402	receptor	Tumor
END	APP	0.662696	ligand	Tumor
END	BMPR2	0.975543	receptor	Tumor
END	CALCRL	0.006265	receptor	Tumor
END	CCL2	0.391902	ligand	Tumor
END	CD34	0.837534	receptor	Tumor
END	CD36	0.588576	receptor	Tumor
END	CD55	0.184108	receptor	Tumor
END	CD99	0.851937	receptor	Tumor
END	CDH5	0.034587	receptor	Tumor
END	COL4A1	0.737987	ligand	Tumor
END	COL4A2	0.72551	ligand	Tumor
END	CXCL12	0.065361	ligand	Tumor
END	CXCL2	0.699485	ligand	Tumor
END	CXCL3	0.294942	ligand	Tumor
END	EDN1	0.358146	ligand	Tumor
END	ENTPD1	0.950192	ligand	Tumor
END	ESAM	0.999994	receptor	Tumor
END	F2R	0.996875	receptor	Tumor
END	HLA-A	0.555645	ligand	Tumor
END	HSPG2	0.675106	ligand	Tumor
END	ICAM1	0.142266	receptor	Tumor
END	ICAM2	0.347015	receptor	Tumor
END	IL6ST	0.995693	receptor	Tumor
END	INSR	0.010012	receptor	Tumor
END	ITGA5	0.999754	receptor	Tumor
END	MCAM	0.996823	receptor	Tumor
END	NOTCH4	0.181808	receptor	Tumor
END	SELE	0.061516	receptor	Tumor
END	TGFBR2	0.004186	receptor	Tumor
END	VCAM1	0.993264	receptor	Tumor
END	VWF	0.687645	ligand	Tumor
T_18T	ANXA1	0.665035	ligand	Tumor
T_18T	AREG	0.922063	ligand	Tumor
T_18T	EREG	0.731488	ligand	Tumor
T_18T	HBEGF	0.529966	ligand	Tumor

T_18T	LAMB3	0.86621	ligand	Tumor
T_18T	LAMC2	0.566191	ligand	Tumor
T_18T	MIF	0.296488	ligand	Tumor
T_18T	NMB	0.556031	ligand	Tumor
T_18T	NMU	0.005094	ligand	Tumor
T_18T	SPP1	0.555359	ligand	Tumor
T_20T	AGT	0.645208	ligand	Tumor
T_20T	CXCL1	0.309881	ligand	Tumor
T_20T	CXCL6	0.994013	ligand	Tumor
T_20T	GDF15	0.968601	ligand	Tumor
T_20T	IGF2	0.869312	ligand	Tumor
T_20T	IL18	0.960998	ligand	Tumor
T_20T	ITGB1	0.364268	receptor	Tumor
T_20T	OCLN	0.987488	receptor	Tumor
T_20T	TNFRSF11B	0.066387	receptor	Tumor
T_23T	AGT	0.465985	ligand	Tumor
T_23T	C3	0.5063	ligand	Tumor
T_23T	CDH2	0.630228	receptor	Tumor
T_23T	CXCL6	0.999041	ligand	Tumor
T_23T	HGF	0.824477	ligand	Tumor
T_23T	HLA-B	0.969892	ligand	Tumor
T_23T	ICAM1	0.993305	receptor	Tumor
T_23T	MDK	0.994719	ligand	Tumor
T_23T	NPW	0.979833	ligand	Tumor
T_23T	NPY1R	0.746866	receptor	Tumor
T_23T	PTPRF	0.573591	receptor	Tumor
T_23T	VCAM1	0.475359	receptor	Tumor
T_24T	AGRN	0.960607	ligand	Tumor
T_24T	APP	0.999296	ligand	Tumor
T_24T	CD44	0.571191	receptor	Tumor
T_24T	CDH11	0.220607	receptor	Tumor
T_24T	COL1A1	0.842983	ligand	Tumor
T_24T	COL4A1	0.756696	ligand	Tumor
T_24T	COL4A2	0.729679	ligand	Tumor
T_24T	COL6A1	0.70866	ligand	Tumor
T_24T	COL6A2	0.999897	ligand	Tumor
T_24T	EGFR	0.01152	receptor	Tumor
T_24T	EPHA2	0.332452	receptor	Tumor
T_24T	FN1	0.999997	ligand	Tumor
T_24T	IGF2	0.197637	ligand	Tumor
T_24T	ITGA2	0.022309	receptor	Tumor
T_24T	ITGA3	0.999952	receptor	Tumor

T_24T	ITGA6	0.40602	receptor	Tumor
T_24T	ITGB4	0.356955	receptor	Tumor
T_24T	ITGB8	1	receptor	Tumor
T_24T	LAMA5	0.051779	ligand	Tumor
T_24T	LAMC2	0.46311	ligand	Tumor
T_24T	PDGFA	0.999743	ligand	Tumor
T_24T	SAA1	0.984696	ligand	Tumor
T_24T	SDC4	0.321337	receptor	Tumor
T_24T	TNC	0.001574	ligand	Tumor
T_24T	TNFRSF1A	0.978243	receptor	Tumor
TIGIT_CD8	CAP1	0.993139	receptor	Tumor
TIGIT_CD8	CD48	0.744565	ligand	Tumor
TIGIT_CD8	CD8A	0.626231	receptor	Tumor
TIGIT_CD8	CD8B	0.999031	receptor	Tumor
TIGIT_CD8	CXCL13	0.307984	ligand	Tumor
TIGIT_CD8	CXCR4	0.902555	receptor	Tumor
TIGIT_CD8	GZMA	0.99982	ligand	Tumor
TIGIT_CD8	HAVCR2	0.588542	receptor	Tumor
TIGIT_CD8	HLA-A	0.749743	ligand	Tumor
TIGIT_CD8	HLA-B	0.821942	ligand	Tumor
TIGIT_CD8	HLA-C	0.763485	ligand	Tumor
TIGIT_CD8	HLA-E	0.999732	ligand	Tumor
TIGIT_CD8	HLA-F	0.569004	ligand	Tumor
TIGIT_CD8	IFNG	0.772216	ligand	Tumor
TIGIT_CD8	IL2RG	0.995671	receptor	Tumor
TIGIT_CD8	ITGB2	0.837769	receptor	Tumor
TIGIT_CD8	KLRC1	0.153706	receptor	Tumor
TIGIT_CD8	LCK	0.777787	ligand	Tumor
TIGIT_CD8	NCL	0.256241	receptor	Tumor
TIGIT_CD8	PTPRC	0.912257	receptor	Tumor
TIGIT_CD8	TNFRSF1B	0.698538	receptor	Tumor
TIGIT_CD8	XCL2	0.575878	ligand	Tumor
VEGFA_MACRO	C3AR1	0.156018	receptor	Tumor
VEGFA_MACRO	C5AR1	0.008711	receptor	Tumor
VEGFA_MACRO	CCL20	0.025665	ligand	Tumor
VEGFA_MACRO	CD36	0.38847	receptor	Tumor
VEGFA_MACRO	CD44	0.616332	receptor	Tumor
VEGFA_MACRO	CD74	0.8774	receptor	Tumor
VEGFA_MACRO	CD86	0.855713	ligand	Tumor
VEGFA_MACRO	CSF1R	0.997422	receptor	Tumor
VEGFA_MACRO	CXCL16	0.6148	ligand	Tumor
VEGFA_MACRO	CXCL2	0.375592	ligand	Tumor

VEGFA_MACRO	CXCL3	0.008753	ligand	Tumor
VEGFA_MACRO	CXCR4	0.856024	receptor	Tumor
VEGFA_MACRO	EREG	0.949843	ligand	Tumor
VEGFA_MACRO	FPR1	1	receptor	Tumor
VEGFA_MACRO	HBEGF	0.638527	ligand	Tumor
VEGFA_MACRO	HCST	0.99956	receptor	Tumor
VEGFA_MACRO	HLA-A	0.705015	ligand	Tumor
VEGFA_MACRO	HLA-B	0.782826	ligand	Tumor
VEGFA_MACRO	HLA-C	0.737404	ligand	Tumor
VEGFA_MACRO	HLA-DQA1	0.907946	ligand	Tumor
VEGFA_MACRO	HLA-DQB1	0.90441	ligand	Tumor
VEGFA_MACRO	HLA-DRB1	1	ligand	Tumor
VEGFA_MACRO	HLA-E	0.979905	ligand	Tumor
VEGFA_MACRO	IFNGR1	0.84088	receptor	Tumor
VEGFA_MACRO	IL1B	0.292496	ligand	Tumor
VEGFA_MACRO	ITGB2	0.999838	receptor	Tumor
VEGFA_MACRO	LGALS9	0.947457	ligand	Tumor
VEGFA_MACRO	NAMPT	0.381893	ligand	Tumor
VEGFA_MACRO	OSM	1	ligand	Tumor
VEGFA_MACRO	PSAP	0.328239	ligand	Tumor
VEGFA_MACRO	PTPRC	0.854003	receptor	Tumor
VEGFA_MACRO	SPP1	0.408392	ligand	Tumor
VEGFA_MACRO	THBS1	0.344107	ligand	Tumor
VEGFA_MACRO	TREM2	0.214936	receptor	Tumor
VEGFA_MACRO	VEGFA	0.276596	ligand	Tumor
CD68_C1QC	AXL	0.130178	receptor	Normal
CD68_C1QC	C3AR1	0.993243	receptor	Normal
CD68_C1QC	CD74	0.630487	receptor	Normal
CD68_C1QC	CD86	0.999995	ligand	Normal
CD68_C1QC	CSF1R	0.236971	receptor	Normal
CD68_C1QC	CXCL12	0.946853	ligand	Normal
CD68_C1QC	CXCL16	0.626379	ligand	Normal
CD68_C1QC	CXCL2	0.317973	ligand	Normal
CD68_C1QC	CXCL3	0.946737	ligand	Normal
CD68_C1QC	GRN	0.999213	ligand	Normal
CD68_C1QC	ICAM1	0.391952	receptor	Normal
CD68_C1QC	IL1B	0.400206	ligand	Normal
CD68_C1QC	NAMPT	0.999996	ligand	Normal
CD68_C1QC	PSAP	0.593675	ligand	Normal
CD68_S100A9	C5AR1	0.316894	receptor	Normal
CD68_S100A9	CD44	0.94025	receptor	Normal
CD68_S100A9	CXCL2	0.983321	ligand	Normal

CD68_S100A9	CXCL3	0.947406	ligand	Normal
CD68_S100A9	FPR1	0.27028	receptor	Normal
CD68_S100A9	IL1B	0.419178	ligand	Normal
CD68_S100A9	NAMPT	0.984034	ligand	Normal
CD68_S100A9	PSAP	0.54183	ligand	Normal
CD8_GZMA	CD8A	0.01606	receptor	Normal
CD8_GZMA	CD8B	0.008944	receptor	Normal
CD8_GZMA	CXCR4	0.187092	receptor	Normal
CD8_GZMA	IFNG	0.909477	ligand	Normal
CD8_GZMA	IL2RG	0.830966	receptor	Normal
CD8_GZMA	KLRB1	0.924526	receptor	Normal
CD8_GZMA	LCK	0.05959	ligand	Normal
CD8_GZMA	PTPRC	0.888724	receptor	Normal
DC	CD74	0.467202	receptor	Normal
DC	CXCL16	0.66331	ligand	Normal
DC	HLA-DQA1	0.665528	ligand	Normal
DC	HLA-DQB1	0.58048	ligand	Normal
DC	HLA-DRB1	0.997982	ligand	Normal
DC_RAMP3	ADM	0.057813	ligand	Normal
DC_RAMP3	APP	0.999855	ligand	Normal
DC_RAMP3	CCL2	0.008797	ligand	Normal
DC_RAMP3	CD36	0.227805	receptor	Normal
DC_RAMP3	CLEC1B	0.666528	ligand	Normal
DC_RAMP3	CXCL2	0.622369	ligand	Normal
DC_RAMP3	F2R	0.994335	receptor	Normal
DC_RAMP3	GDF15	0.051588	ligand	Normal
DC_RAMP3	ICAM1	0.987297	receptor	Normal
DC_RAMP3	IL6ST	0.88371	receptor	Normal
DC_RAMP3	INSR	0.309696	receptor	Normal
DC_RAMP3	ITGB1	0.975566	receptor	Normal
DC_RAMP3	KDR	0.143692	receptor	Normal
DC_RAMP3	LIFR	0.989718	receptor	Normal
DC_RAMP3	NRP1	0.064892	receptor	Normal
DC_RAMP3	TGFBR2	0.985851	receptor	Normal
END	APP	0.641643	ligand	Normal
END	CCL2	0.957142	ligand	Normal
END	CD36	0.57942	receptor	Normal
END	CXCL2	0.984355	ligand	Normal
END	HSPG2	0.867246	ligand	Normal
END	IL6ST	0.999626	receptor	Normal
END	INSR	0.005206	receptor	Normal
END	ITGB1	0.983286	receptor	Normal

END	TGFBR2	0.996013	receptor	Normal
END	VWF	0.047824	ligand	Normal
HPE_1	MIF	1	ligand	Normal
HPE_1	SAA1	0.98614	ligand	Normal

Table S4. Cell type interaction scores calculated by eLBP algorithm.

Ligand	Receptor	Interaction Score	Type
CAF	CCR7_T	0.761605	Tumor
CAF	T_24T	0.560202	Tumor
CAF	VEGFA_MACRO	0.680335	Tumor
END	CCR7_T	0.557263	Tumor
END	END	0.862893	Tumor
T_18T	VEGFA_MACRO	0.588971	Tumor
T_24T	CCR7_T	0.64026	Tumor
T_24T	T_24T	0.563989	Tumor
T_24T	VEGFA_MACRO	0.645288	Tumor
TIGIT_CD8	TIGIT_CD8	0.563293	Tumor
VEGFA_MACRO	TIGIT_CD8	0.571811	Tumor
CD68_C1QC	CD8_GZMA	0.173599	Normal
CD68_C1QC	DC_RAMP3	0.299383	Normal
CD68_S100A9	DC_RAMP3	0.293443	Normal
DC_RAMP3	CD68_C1QC	0.625614	Normal
DC_RAMP3	CD8_GZMA	0.605967	Normal
DC_RAMP3	DC	0.463848	Normal
END	CD68_C1QC	0.399777	Normal
END	DC	0.296459	Normal

Table S5. Interaction scores of gene pairs among the cell types calculated by eLBP algorithm.

Ligand Cluster	Receptor Cluster	Interaction pairs	Interaction Scores	Type
CAF	CCR7_T	FN1_CD44	0.75929	Tumor
CAF	CCR7_T	COL1A1_CD44	0.763919	Tumor
CAF	T_24T	FN1_CD44	0.558588	Tumor
CAF	T_24T	COL1A1_CD44	0.561816	Tumor
CAF	VEGFA_MACRO	FN1_CD44	0.598488	Tumor
CAF	VEGFA_MACRO	COL1A1_CD44	0.6027	Tumor
CAF	VEGFA_MACRO	APP_CD74	0.839818	Tumor

END	CCR7_T	COL4A1_CD44	0.562916	Tumor
END	CCR7_T	COL4A2_CD44	0.551611	Tumor
T_18T	VEGFA_MACRO	ANXA1_FPR1	0.65267	Tumor
T_18T	VEGFA_MACRO	LAMB3_CD44	0.525272	Tumor
T_24T	CCR7_T	FN1_CD44	0.763801	Tumor
T_24T	CCR7_T	COL1A1_CD44	0.643339	Tumor
T_24T	CCR7_T	COL4A1_CD44	0.57464	Tumor
T_24T	CCR7_T	COL4A2_CD44	0.55626	Tumor
T_24T	CCR7_T	COL6A1_CD44	0.53842	Tumor
T_24T	CCR7_T	COL6A2_CD44	0.765101	Tumor
T_24T	VEGFA_MACRO	FN1_CD44	0.602598	Tumor
T_24T	VEGFA_MACRO	COL1A1_CD44	0.508525	Tumor
T_24T	VEGFA_MACRO	COL6A2_CD44	0.603783	Tumor
T_24T	VEGFA_MACRO	APP_CD74	0.866246	Tumor
VEGFA_MACRO	TIGIT_CD8	LGALS9_HAVCR2	0.538534	Tumor
VEGFA_MACRO	TIGIT_CD8	HLA-E_CD8A	0.605088	Tumor
CD68_C1QC	CD8_GZMA	CXCL12_CXCR4	0.173599	Normal
CD68_C1QC	DC_RAMP3	NAMPT_INSR	0.299383	Normal
CD68_S100A9	DC_RAMP3	NAMPT_INSR	0.293443	Normal
DC_RAMP3	CD68_C1QC	APP_CD74	0.625614	Normal
DC_RAMP3	CD8_GZMA	CLEC1B_KLRB1	0.605967	Normal
DC_RAMP3	DC	APP_CD74	0.463848	Normal
END	CD68_C1QC	APP_CD74	0.399777	Normal
END	DC	APP_CD74	0.296459	Normal

Table S6. Critical TFs participated in the cell communication among the cell types.

TF	TF_combind_score	mean_TF	Database	Cell Type	Tissue Type
KLF2	0.93	0.903021	ChEA3	CD68_C1QC	Normal
**STAT1**	0.79	0.984897	ENCODE	CD68_C1QC	Normal
**EGR1**	0.75	0.978537	ENCODE	CD68_C1QC	Normal
JUN	0.81	3.227345	ENCODE	CD68_C1QC	Normal
**JUND**	0.81	1.443561	ENCODE	CD68_C1QC	Normal
FOS	0.8	10.14944	ENCODE	CD68_C1QC	Normal
ATF3	0.84	1.855805	ChEA3	DC_RAMP3	Normal
**KLF4**	0.82	1.262172	ChEA3	DC_RAMP3	Normal
EGR1	0.85	1.531835	ChEA3	DC_RAMP3	Normal
SOX17	0.91	1.048689	ChEA3	DC_RAMP3	Normal

**STAT3**	0.83	0.827715	ChEA3	DC_RAMP3	Normal
FOS	0.83	7.588015	ENCODE	DC_RAMP3	Normal
JUN	0.82	11.99064	ENCODE	DC_RAMP3	Normal
MEF2C	0.87	1.537453	ENCODE	DC_RAMP3	Normal
H2AFZ	0.82	1.046816	ENCODE	DC_RAMP3	Normal
**SPI1**	0.85	3.004563	ChEA3	VEGFA_MACRO	Tumor
NUCKS1	0.81	1.720991	ChEA3	VEGFA_MACRO	Tumor
CEBPB	0.84	5.097784	ChEA3	VEGFA_MACRO	Tumor
NFE2L2	0.89	2.559974	ChEA3	VEGFA_MACRO	Tumor
**SPI1**	0.87	3.004563	ENCODE	VEGFA_MACRO	Tumor
STAT3	0.83	1.08279	ENCODE	VEGFA_MACRO	Tumor
STAT1	0.84	1.593872	ENCODE	VEGFA_MACRO	Tumor
FOS	0.84	13.12256	ENCODE	VEGFA_MACRO	Tumor
CEBPB	0.86	5.097784	ENCODE	VEGFA_MACRO	Tumor
CHD1	0.83	1.22751	ENCODE	VEGFA_MACRO	Tumor
H2AFZ	0.86	2.777705	ENCODE	VEGFA_MACRO	Tumor
JUND	0.86	2.984355	ENCODE	VEGFA_MACRO	Tumor
MEF2C	0.84	1.085398	ENCODE	VEGFA_MACRO	Tumor
CHD2	0.84	0.945893	ENCODE	VEGFA_MACRO	Tumor
EGR1	0.84	1.511734	ENCODE	VEGFA_MACRO	Tumor
JUN	0.84	6.292699	ENCODE	VEGFA_MACRO	Tumor
ELF1	0.85	1.490874	ENCODE	VEGFA_MACRO	Tumor
**IRF1**	0.83	1.276402	ENCODE	VEGFA_MACRO	Tumor
**IRF1**	0.82	1.276402	TRANSFAC	VEGFA_MACRO	Tumor
ETS2	0.81	1.236636	TRANSFAC	VEGFA_MACRO	Tumor
ELF1	0.79	1.490874	TRANSFAC	VEGFA_MACRO	Tumor
REL	0.86	4.304433	TRANSFAC	VEGFA_MACRO	Tumor

Table S7. 54-gene classifiers

Gene	Gene	Gene
IRF1	PTPRC	NCF2
STX11	PLAUR	C1QA
TYROBP	ACP5	ARHGDIB
CYBB	RAP1A	CSF1R
EREG	SOCS3	CXCR4
GABARAP	EIF4A3	CD74
ISG15	IFNGR1	FCER1G
OSM	CORO1A	ITGB2
VAMP8	SRGN	CD14
LGALS9	FPR1	HCK

CYBA	AP1S2	LYZ
C1QC	RGS10	CD86
RHOA	SLC2A3	RGS1
SPI1	LCP1	VCAN
LY96	CEBPB	HCLS1
CST3	HCST	CD83
FCGR2A	MAP3K8	CD68
ASAH1	NR4A2	TREM1
